# Stress granule-related genes during embryogenesis of an invertebrate chordate

**DOI:** 10.3389/fcell.2024.1414759

**Published:** 2024-08-01

**Authors:** Laura Drago, Alessandro Pennati, Ute Rothbächer, Ryuji Ashita, Seika Hashimoto, Ryota Saito, Shigeki Fujiwara, Loriano Ballarin

**Affiliations:** ^1^ Department of Biology, University of Padova, Padua, Italy; ^2^ Institute of Zoology, University of Innsbruck, Innsbruck, Austria; ^3^ Department of Chemistry and Biotechnology, University of Kochi, Kochi, Japan

**Keywords:** ascidian, embryogenesis, stress granules, TIAR, TTP, G3BP

## Abstract

Controlling global protein synthesis through the assembly of stress granules represents a strategy adopted by eukaryotic cells to face various stress conditions. TIA 1-related nucleolysin (TIAR), tristetraprolin (TTP), and Ras-GTPase-activating protein SH3-domain-binding protein (G3BP) are key components of stress granules, allowing the regulation of mRNA stability, and thus controlling not only stress responses but also cell proliferation and differentiation. In this study, we aimed at investigating the roles of *tiar*, *ttp*, and *g3bp* during embryogenesis of the solitary ascidian *Ciona robusta* under both physiological and stress conditions. We carried out CRISPR/Cas9 to evaluate the effects of gene knockout on normal embryonic development, and gene reporter assay to study the time and tissue specificity of gene transcription, together with whole-mount *in situ* hybridization and quantitative real time PCR. To induce acute stress conditions, we used iron and cadmium as “essential” and “non-essential” metals, respectively. Our results highlight, for the first time, the importance of *tiar, ttp*, and *g3bp* in controlling the development of mesendodermal tissue derivatives during embryogenesis of an invertebrate chordate.

## 1 Introduction

To store energy for maintaining normal cellular homeostasis, eukaryotic cells have evolved sophisticated strategies that involve the attenuation of global protein synthesis through the formation of stress granules (SGs). The dynamic assembly of these membraneless foci in the cytoplasm, mediated by the overexpression of mRNA-binding proteins, allows the temporary termination of translation initiation of selected mRNAs confined inside SGs to prepare the cell for future acute stress conditions when mRNAs are rapidly released from SGs and translated into the required proteins (Arribere et al., 2011; Liu and Qian, 2014; Protter and Parker, 2016; Somasekharan et al., 2020). SGs consist of a core structure of key mRNA-binding proteins and mRNAs that are connected by strong interactions, surrounded by a less concentrated and more dynamic shell. In this shell, proteins and mRNAs can be easily exchanged with other types of cytoplasmic foci, such as processing bodies (p-bodies) (García-Mauriño et al., 2017), where mRNAs undergo degradation, germ cell granules (Mukherjee and Mukherjee, 2021), for maternal mRNA storage during early development, and neuronal granules (Söhnel and Brandt, 2023), important for synaptic remodeling. Therefore, the regulation of mRNA stability plays a key role not only in stress responses but also in cell proliferation, differentiation, and development.

TIA 1-related nucleolysin (TIAR) and Ras-GTPase-activating protein SH3-domain-binding protein (G3BP) are components of the SG core, whereas tristetraprolin (TTP) is a shell element. These proteins can recognize and bind AU-rich elements of mRNAs codifying for proteins involved in cell growth and differentiation, signal transduction, apoptosis, nutrient transport, and metabolism (e.g. tumor necrosis factor-alpha (*tnf-α*)*, p38,* signal transducer and activator of transcription 5b (*stat5b*)*,* and *myc*) at either their 3′ or 5′ untranslated regions (UTRs): in the first case, the interaction functions as a deadenylation signal (Gallouzi et al., 1998; Piecyk et al., 2000; Dean et al., 2004; Vignudelli et al., 2010; Geng et al., 2015; Makita et al., 2021). Post-transcriptional control operated by SGs is facilitated by the presence of RNA-recognition motifs (RRMs) in TIAR and G3BP, and CCCH tandem zinc finger domains in TTP (Cléry et al., 2008; von Roretz et al., 2011).

Mammalian TIAR has three RRMs: RRM1 confers the ability to interact with other RNA-binding proteins, RRM2 is the main DNA/RNA interaction motif, and RRM3 regulates TIAR export from the nucleus to the cytoplasm (Waris et al., 2014). A detailed description of TIAR can be found in our previous study (Drago et al., 2023). The importance of TIAR during embryogenesis has been documented in mice (Kharraz et al., 2010; Sánchez-Jiménez and Izquierdo, 2013; Liu et al., 2023), where both under- and overexpression of the gene is associated with impaired embryo formation or lethality, and in *Caenorhabditis elegans* (Huelgas-Morales et al., 2016), where it is involved in the protection of the germline from heat stress. In addition, *tiar* knockout causes infertility and impaired gametogenesis in adult mice (Beck et al., 1998; Piecyk et al., 2000).

G3BP is a site-specific ribonucleic acid endonuclease that is able to bind the SH3 domain of Ras-GTPase-activating protein in serum-stimulated cells, as firstly reported by Parker et al. (1996). G3BP phosphorylation sites modulate its catalytic activity. For example, in proliferating cells, G3BP is hypophosphorylated, leading to the loss of its ability to cleave AU-rich mRNA. In mammals, G3BP contains one carboxyl C-terminal RRM, with conserved hydrophobic amino acids in the ribonucleoprotein 1 (RNP1; eight amino acids) and RNP2 (six amino acids) sequences, both essential for the interaction with RNA, one N-terminal nuclear transport factor 2 (NTF2) domain, associated with G3BP nuclear translocation and dimerization by mediating the Ras-GDP nuclear import through nucleoporins, and one N-terminal arginine-glycine-rich region, which can be easily methylated allowing the regulation of SG disassembly. Conversely, the C-terminal region of G3BP can induce the phosphorylation of eukaryotic initiation factor 2 alpha, which reduces the availability of the initiation factor 2 alpha–GTP–tRNAMet ternary complex necessary to initiate protein translation, promoting SG assembly (Kang et al., 2021; Tourrière et al., 2023).

Three homologous G3BPs are known in mammals: G3BP1, G3BP2a, and G3BP2b. G3BP1 differs from G3BP2 by a substitution (valine with isoleucine) in RNP2, and G3BP2a lacks 33 amino acids in its central proline-rich region compared with G3BP2b. Proline-rich motifs (PxxP) are required for the activation of protein kinase, which is important for the nucleation of SGs: G3BP1 has only one PxxP motif, whereas G3BP2a and G3BP2b have four and five PxxP motifs, respectively (Kang et al., 2021). G3BP2s contain more arginine residues in the arginine-glycine-rich region than G3BP1, which may lead to different target specificities of mRNAs and make G3BP2s more prone to oligomerization. In mammals, G3BP1 is highly expressed in the lungs and kidneys, while G3BP2s are highly expressed in the small intestine and brain (Kennedy et al., 2001). G3BP1 and G3BP2 can also form homo- or heterodimers through their NTF2 domain, thus explaining their coexistence in SGs, since the dysfunction of one of them can negatively affect the other (Jin et al., 2022). The NTF2 domain plays an important role in viral replication as it can bind viral motifs, allowing G3BP recruitment by the viral replication complex to evade the cellular immunity of the host (Götte et al., 2019; Wang and Merits, 2022). In mouse embryogenesis, the inactivation of G3BP leads to embryonic lethality or growth retardation and neonatal lethality (Zekri et al., 2005).

TTP, also known as TIS11 and ZFP36, promote poly (A) tail removal or deadenylation in AU-rich mRNAs, leading to their conservation or degradation in SGs and p-bodies, respectively, with partial or complete relocalization from p-bodies to SGs in stressed cells (Kedersha et al., 2005). The activation of TTP was first observed by Varnum et al. (1989) in murine Swiss 3T3 cells as a primary response to the tumor promoter tetradecanoyl phorbol acetate. TTP translocates from the nucleus to the cytoplasm upon stimulation with a mitogen (Taylor et al., 1996). Two or more zinc finger domains, able to bind Zn^2+^ with high affinity, have been identified in TTP from mammals, *Xenopus*, *Drosophila,* and yeast; they allow self-regulation of the protein by destabilizing its own mRNA. In addition, the tetradecanoyl phorbol acetate-inducible sequence 11 (TIS11) domain is involved in calcium signaling-induced apoptosis of B cells (Murata et al., 2005). In mammals, four homologous TTPs are known, the knockout of which causes systemic inflammatory syndrome in mice due to the chronic increase of TNFα levels and embryonic lethality. TTP negatively regulates hematopoietic and erythroid cell differentiation by decreasing STAT5B mRNA expression (Vignudelli et al., 2010). It is implicated in maternal mRNA turnover in mouse embryogenesis, and gene disruption leads to female infertility in mice (Ramos et al., 2004). The protein is also important for yolk sac and placental development (Stumpo et al., 2016). TTP plays a key role in regulating immune function and cell growth through mRNA deadenylation or degradation. It can act as tumor suppressor by inhibiting cell proliferation in breast cancer cells (Huang et al., 2016), and it can regulate apoptotic events, through the modulation of the inflammatory-mediator TNF-α in macrophages (Johnson and Blackwell, 2002; Holmes et al., 2012).

TIAR, G3BP, and TTP have been widely studied as molecular markers of SGs in vertebrates. In marine invertebrates, the only information on molecular markers of SGs was found in our previous studies, in which we investigated *tiar* and *ttp* expression during metal-induced stress conditions in adults of the solitary ascidian *Ciona robusta* (Drago et al., 2021), and the role of *g3bp, tiar,* and *ttp* during non-embryonic development of the colonial ascidians *Botryllus schlosseri* and *B. primigenus* (Drago et al., 2023; 2024), characterized by cyclical generation changes. In the case of *B. schlosseri,* the involvement of TIAR protein in the colonial blastogenetic cycle was also investigated (Drago et al., 2023). In the present study, we aimed at elucidating the functions of *tiar*, *ttp*, and *g3bp* during the early development of *C. robusta* under physiological and stress conditions. We used clustered regularly interspaced short palindromic repeats (CRISPR)/Cas9 technique to study the effects of *tiar*, *g3bp*, and *ttp* knockout on normal embryo development, and gene reporter assay to study time and cellular specificity of gene transcription, reflecting the action of the regulatory sequences in embryos under control and stress conditions, the latter due to exposure to iron and cadmium, as “essential” and “non-essential” metals, respectively. *tiar*, *g3bp.* and *ttp* transcription was analyzed using whole-mount *in situ* hybridization (ISH) and quantitative realtime PCR (qRT-PCR).

## 2 Materials and methods

### 2.1 Animals

*C. robusta* is an invertebrate chordate belonging to the subphylum Tunicata that is widely used in embryogenetic studies because it allows obtaining hundreds of embryos after a single artificial fertilization event, which develop up to the larval stage within a day, larvae can then adhere to a solid substrate and metamorphose into adults (Passamaneck and Di Gregorio, 2005; Hotta et al., 2007; Hotta et al., 2020). In addition, *Ciona* embryos can be easily electroporated using specific constructs for mutagenic experiments (Stolfi et al., 2014; Fujiwara and Cañestro, 2018; Zeller, 2018; Papadogiannis et al., 2022). *Ciona* is an excellent model organism because of its annotated compact genome, which is easily accessible in the ANISEED database (https://www.aniseed.fr/), with relatively short intergenic regions that facilitate the identification of *cis*-regulatory elements for the control of gene expression (Dardaillon et al., 2020).

### 2.2 Oligonucleotide design

Oligonucleotides for gene expression studies (qRT-PCR) and gene knockout experiments (CRISPR/Cas9) were designed in the coding region (CDS) of *tiar* (transcript ID: KY2019:KY.Chr7.535.v1.SL2-1), *ttp* (transcript ID: KY2019:KY.Chr4.13.v2.nonSL9-1), and *g3bp* (transcript ID: KY2019:KY.Chr1.2038.v1.SL1-1), obtained from the ANISEED database. The final sequence was confirmed by amplicon sequencing (Eurofins Genomics Europe Shared Services GmbH, Ebersberg, Germany). The intron/exon compositions were then analyzed (http://ghost.zool.kyoto-u.ac.jp/SearchGenomekh.html). Oligonucleotides for the preparation of single guide RNAs (sgRNAs) were designed using CRISPOR (Concordet and Haeussler, 2018) with a Doench’16 cutoff ≥60. To obtain an efficient knockout, we placed sgRNAs in the genomic areas covering the functional domains. Off-target sequences with less than three mismatches were avoided as well as sequences with four or more consecutive thymine near the 3′ end, as they can cause termination of transcription by RNA polymerase III. Oligos containing sgRNA sequences were ordered with overhangs complementary to the vector sequence (U6>sgRNA F + E, addgene59986) and to initiate the transcription of sgRNAs at the U6 promoter (Stolfi et al., 2014). Primers for validating genomic DNA cleavage achieved by the CRISPR/Cas9 constructs were designed to flank the target locus so that the potential cleavage site was not in the center of the amplicon.

For the gene reporter assay, primers were designed to isolate potential *tiar*, *ttp*, and *g3bp cis*-regulatory regions identified in the ghost database genome browser (http://ghost.zool.kyoto-u.ac.jp/default_ros.html) in areas of high chromatin accessibility (staged, whole-embryo wild-type ATAC-seq data) and upstream of the transcription start site. The IDT OligoAnalyzer tool (https://eu.idtdna.com/pages/tools/oligoanalyzer) was used to check all designed primers synthesized by Merck Life Science S.r.l. (Milan, Italy), Eurofins Genomics Japan (Tokyo), and Microsynth (Balgach, Switzerland) (Supplementary Table S1).

### 2.3 Gene and protein organization of *g3bp*

The gene structure of *C. robusta g3bp* was analyzed by matching the cDNA, in part obtained by sequencing and then reconstructed *in silico* using the ANISEED database, with the genomic sequence (http://ghost.zool.kyoto-u.ac.jp/SearchGenomekh.html). For sequence comparison studies and to check the correct name of the analyzed sequence of *C. robusta*, we used the NCBI BLAST tool (https://blast.ncbi.nlm.nih.gov/Blast.cgi). To study the G3BP protein organization, orthologous amino acid sequences of some metazoans were aligned with those of *C. robusta* using Clustal Omega software (https://www.ebi.ac.uk/Tools/msa/clustalo/). The domain architecture previously predicted using the SMART program (http://smart.embl-heidelberg.de/smart/set_mode.cgi?NORMAL=1) was visualized. The LALIGN tool (https://www.ebi.ac.uk/Tools/psa/lalign/) was used to compare G3BP sequence of *C. robusta* with each orthologous sequence considered in the multi-alignment analysis to evaluate the degree of identity and similarity. G3BP protein molecular weight was calculated with Expasy Compute pI/Mw tool (https://web.expasy.org/compute_pi/).

### 2.4 Plasmid preparation

The In-Fusion primer design tool (Takarabio.com) was used to design and simulate cloning procedures. *Cis*-regulatory regions of *tiar* (530 nucleotides (nt) upstream of CDS), *ttp* (318 nt upstream of CDS), and *g3bp* (1500 nt upstream of CDS), selected with primers containing specific overhangs (Supplementary Table S1), were introduced into the pSP72-1.27 vector driving LacZ (Corbo et al., 1997), through a ligation reaction performed with the In-Fusion Snap Assembly (Takara Bio, Japan) master mix (50°C for 15 min). Q5 High-Fidelity polymerase (NEB) was used to linearize the backbone vector (LaZ_IF_Fw and LacZ_IF_Rev primers, Supplementary Table S1) and potential cis-regulatory regions, starting from genomic DNA extracted from sperm (see paragraph below). PCR products were gel-purified using a Monarch DNA Gel Extraction Kit (NEB) and used for ligation reactions. The ligation products were cloned into JM109 competent cells (Promega, United States) and screened by colony PCR using OneTaq Quick-Load 2X Master Mix (NEB). Positive colonies were cultured overnight (o/n) for miniprep, performed according to the PureYield Plasmid Miniprep System (Promega) instructions, and confirmed by sequencing (Microsynth, Switzerland). The three constructs were then cultured for 24 h at 37°C and purified with NucleoBond Xtra Midi Kit (Macherey–Nagel, Germany) and used for gene reporter assay.

In the present study, we produced ten sgRNA vectors targeting *tiar*, *ttp*, and *g3bp*, to guide the Cas9 nuclease, according to Stolfi et al. (2014). U6>sgRNA (F + E) purified plasmid, linearized with *Bsa*I (New England Biolabs, Japan), was ligated with double-stranded DNA (Supplementary Table S1) (vector to insert ratio 1:2), annealed at 10 µM (90°C for 5 min, 30°C for 30 min), using T4 DNA ligase (Promega), o/n at 4°C.

For purification of digested U6>sgRNA (F + F + E), the gel slice was dissolved in NaI solution (gel slice-to-NaI ratio 1:3) containing glass powder. Once the gel was completely dissolved by mixing, EasyTrap buffer (50% ethanol, 100 mM NaCl, and 10 mM Tris-HCl, pH 7.5) and 1 mM EDTA were used to wash the glass powder. Then, DNA was separated from glass powder by heating at 55°C in nuclease-free water and centrifugation. Ligation products were cloned in DH-5α *Escherichia coli* chemically competent cells, and plasmids were purified from positively screened colonies with QIAprep Miniprep Kit (QIAGEN) and sequenced at the Research Institute for Molecular Genetics of Kōchi University. Therefore, the constructs were cloned again into TOP10 competent cells, which were cultured for 24 h, purified using the QIAGEN Plasmid Midi Kit, and used for CRISPR/Cas9 experiments.

The FoxD > Cas9 plasmid was prepared from the FoxD > LacZ plasmid (Mita and Fujiwara, 2007). The primers used to amplify the upstream region of *FoxD* using Takara Ex Premier DNA Polymerase (Takara Bio) are listed in Supplementary Table S1. EF1 α > Cas9 (addgene59987) (Stolfi et al., 2014) was digested with *NheI* (NEB) and *SnaBI* (NEB) to excise out the upstream region of EF1α, then the PCR-amplified *FoxD* upstream region and restriction-digested plasmid vector were gel-purified, using the EasyTrap buffer as described above, and finally fused by a recombination reaction using the In-Fusion HD Cloning Kit (Takara Bio). The In-Fusion reaction solution was used to transform the TOP10 strain of *E. coli*.

### 2.5 Genomic DNA extraction

Genomic DNA was extracted from *Ciona* sperm. SDS lysis buffer (1% SDS, 50 mM NaCl, 10 mM EDTA, and 50 mM Tris, pH 8) with 0.02 mg/mL of proteinase XIV was mixed with the sperm o/n at 50°C. Phenol-chloroform-isoamyl alcohol (25:24:1) was added to remove digested proteins, followed by centrifugation at 12,000 × *g* for 45 min, and 3 M sodium acetate (pH 5.2) was added to the supernatant together with absolute ethanol (EtOH). After DNA precipitation o/n at −20°C and centrifugation at 12,000 × *g* for 20 min, DNA pellet was washed with 80% EtOH and centrifugated twice at 12,000 × *g* for 20 min. The DNA pellet was then air-dried and resuspended in nuclease-free water. The DNA concentration and purity were assessed using a Nanodrop ND-1000 spectrophotometer (Thermo Fisher Scientific).

### 2.6 Embryo collection and treatment

Adult specimens of *C. robusta* were collected in Chioggia, in the southern area of the Lagoon of Venice, Italy, and in the Usa area of Tosa city, Kōchi Prefecture, Japan; in the last case, *Ciona* juveniles, kindly provided by Yutaka Satou Lab of Kyoto University, were reared in nature until sexual maturation. Animals were maintained in aerated aquaria, filled with filtered sea water (FSW) or Super Marine Art SF1 artificial sea water (ASW; Tomita Pharmaceuticals), at 18°C under direct light and fed with Phyto Marine (Oceanlife, Bologna, Italy) and Reefpearls (DVH Aquatic, Holland).

Good-quality eggs and sperms from dissected adults have been used for *in vitro* fertilization (Kari et al., 2016). Sperm was activated with a solution of 0.05 M Tris (pH 9.5) in FSW or ASW and added to the eggs. After 15 min, embryos were gently collected, dechorionated with dechorionation solution (1% sodium thioglycolate and 0.05% Proteinase XIV) and 0.05 M NaOH in FSW or ASW and washed in FSW or ASW. Approximately 200 embryos were reared in 10-cm agarose-coated Petri dishes to prevent the adhesion of the embryos and cultured at 18°C in FSW or ASW with or without (controls) metal ions. The samples were collected at the desired stage and subsequently used for whole-mount ISH and qRT-PCR. For metal treatments, iron (Fe^2+^) was used as an essential metal, and cadmium (Cd^2+^) was used as a non-essential metal; after several tests to determine the lethal dose, 50%, 1 µM of metal ions was chosen as sub-lethal concentration. Storage solutions were previously prepared by dissolving the metal salt (FeCl_2_ or CdCl_2_) in distilled water, and the metal concentration was verified with a Perkin Elmer 4,000 Atomic Absorption Spectrophotometer; working solutions were prepared by diluting the storage solutions in FSW.

In another series of experiments (gene reporter and CRISPR/Cas9), dechorionated embryos were immediately electroporated.

### 2.7 Electroporation

Dechorionated embryos were electroporated with a single pulse at 50 V for 16 ms in a 4-mm electroporation cuvette, containing 0.77 M mannitol solution (Kari et al., 2016). For the gene reporter assay, we used 40 μg of plasmid, containing *tiar* (pTiar > LacZ), *ttp* (pTtp > LacZ), or *g3bp* (pG3bp > LacZ) cis-regulatory sequences, driving LacZ, and 30 μg of Fog > H2B:mCherry plasmid, marking the successful uptake of the electroporation mix, in a total volume of 350 µL (Papadogiannis et al., 2022). For CRISPR/Cas9 experiments, we used 75 μg of sgRNA-expressing plasmids for *tiar*, *ttp*, or *g3bp* knockout (U6 > tiar_sgRNA, U6 > ttp_sgRNA, and U6 > g3bp_sgRNA) and 25 μg of Eef1a > Cas9 or FoxD > Cas9 plasmids. Electroporated plasmid amounts were per 700 μL of total volume.

For the gene reporter assay, electroporated embryos were reared in FSW with or without metal ions, as described above, until the desired stage, whereas for CRISPR/Cas9 experiments, electroporated embryos were maintained in ASW until the desired stage. Eef1a > Cas9 was used to evaluate CRISPR/Cas9-targeted mutagenesis of *tiar*, *ttp*, and *g3bp* loci at the initial tailbud stage, considering that the ubiquitous Eef1a promoter is not active before the 64 cell stage (Stolfi et al., 2014), using a GeneArt Genomic Cleavage Detection Kit. Once sgRNA functions in gene knockout, FoxD > Cas9 was used in electroporation for tissue-specific (mesendodermal derivatives, including the posterior brain and central nervous system, trunk endoderm, notochord, and trunk ventral cells) mutagenesis of *Ciona* embryos (Imai et al., 2009; Abitua et al., 2012; Gandhi et al., 2017).

### 2.8 Gene reporter assay

Gene reporter assay was carried out to analyze the differential tissue activity of cis-regulatory elements in embryos developed in the presence of metals. The following developmental stages were considered: mid-gastrula, mid-tailbud II, and hatching larva. The transcriptional profiles of the three considered genes at the studied developmental stages were deduced from the RNAseq datasets presents in ANISEED database. These developmental stages are marked by the appearance of peculiar and key features. In mid-gastrula, the neural plate starts to form. In mid-tailbud II, a clear distinction between tail and trunk can be observed, the closure of the neuropore is completed, the otolith and ocellus pigmentation appears and the notochord vacuolization begins. These two stages lack the external tunic which increases the background signal in whole-mount ISH experiments. The hatching larva is a well-defined developmental stage with formed anterior papillae, useful for adhesion to the substrate prior to metamorphosis into adult, and tail movements (Hotta et al., 2007).

Samples were incubated for 1 h in blocking solution (0.25% tergitol, 5% heat-inactivated normal goat serum in phosphate-buffered saline (PBS: 1.37 M NaCl, 0.03 M KCl, 0.015 M KH_2_PO_4_, and 0.065 M Na_2_HPO_4_, pH 7.2), and incubated with anti-beta-galactosidase purified monoclonal antibody (Promega), 1:200 in blocking solution for 2 h. After several washes with 0.25% Tergitol in PBS, the embryos were incubated in Alexa Fluor 488 goat anti-mouse IgG (H + L) (Invitrogen), at 1:500 in blocking solution for 1 h. Embryos were washed, mounted in Vectashield (Vector Laboratories), and observed under a Leica TCS SP5 Confocal microscope.

### 2.9 CRISPR/Cas9 knockout: DNA cleavage detection assay and phenotype study

DNA cleavage validation for *tiar*, *ttp*, and *g3bp* was assessed using the GeneArt Genomic Cleavage Detection Kit (Thermo Fisher Scientific), starting from genomic DNA extracted from initial tailbud embryos co-electroporated with specific sgRNAs and Eef1a > Cas9 before the first cell division. Following cleavage, indels were created in genomic DNA by normal cellular repair mechanisms; therefore, the loci where double-strand breaks were expected were PCR-amplified (primers in Supplementary Table S1). The PCR products were denatured and reannealed, and mismatches were subsequently detected and cleaved using a Detection Enzyme kit. Negative controls were also considered for each sgRNA by adding nuclease-free water instead of the detection enzyme. The resultant bands were visualized using electrophoresis and analyzed using ImageJ software. The absence of cleavage was also observed in embryos electroporated with Eef1a > Cas9 alone.

For each gene, at least one sgRNA vector was identified as active in DNA cleavage and used in the CRISPR/Cas9 experiments co-electroporated with FoxD > Cas9 to mediate tissue-specific mutagenesis and evaluate the importance of TIAR, TTP, and G3BP for correct embryo development. Electroporated embryos were fixed at the desired stage (mid-tailbud II and hatching larva) in 2% paraformaldehyde in ASW with 0.1% Tween-20 for 15 min and blocked in 1% powdered milk in PBS containing 0.1% Tween-20 (PBST) for 30 min. Embryos were treated with 100 nM Rhodamine Phalloidin (Thermo Fisher Scientific) or Acti-stain 488 fluorescent Phalloidin (Cytoskeleton, Inc.) in PBST for 20 min in dark conditions, followed by 1 μg/mL DAPI (Thermo Fisher Scientific) or Hoechst (Life Technologies Corporation) in PBST for 15 min. Embryos were mounted in 86% glycerol and observed under a Leica TCS SP5 or Nikon C1si confocal microscope. Embryos electroporated with FoxD > Cas9 alone were considered as controls for normal phenotype development in the absence of DNA cleavage.

### 2.10 Whole-mount ISH

Specific DNA templates were obtained from cDNA libraries (VES103-I09 for TIAR, VES72-L12 for G3BP, and VES65-B17 for TTP) (Roure et al., 2007). Kanamycin-resistant colonies were selected, cultured o/n, purified using the Plasmid Miniprep system (Promega), and verified by sequencing (Microsynth, Switzerland). PCR products with flanking T7 promoters were used as templates for the synthesis of antisense riboprobes using T7 RNA polymerase (Roche) (Mercurio et al., 2023). Riboprobes were purified by precipitation with 0.1 M LiCl and 75% EtOH. After centrifugation at 12,000 x g for 15 min, the pellet was washed with 70% EtOH, air-dried, and dissolved in nuclease-free water.

To verify the correlation between *tiar*, *ttp*, and *g3bp* gene expression and gene reporter assay results, whole-mount ISH was carried out at the mid-gastrula and mid-tailbud II stages, as previously described (Mercurio et al., 2021). Briefly, control and metal-treated embryos, were fixed (4% paraformaldehyde, 0.5 M NaCl in 0.1 M MOPS) for 1 h, dehydrated with EtOH, and stored in 70% EtOH at −20°C until use. After rehydration in EtOH, embryos were permeabilized with 2 μg/mL proteinase K in PBST at 37°C for 5 min, fixed again for 1 h, and pre-hybridized in hybridization solution, i.e. 50% formamide, sodium citrate solution (SSC: 0.6 M NaCl, 0.068 M sodium citrate, pH 4.5), 1 μg/mL yeast tRNA, 0.25 μg/mL heparin, and 0.1% Tween-20 in nuclease-free water, at 52°C for 2 h. Hybridization was carried out at 52°C for 18 h. After several washes (50% formamide, SSC, and 0.1% Tween-20 in nuclease-free water) at 52°C, embryos were incubated in 25% normal goat serum in PBST for 2 h. Incubation with anti-digoxigenin-AP Fab antibody (Roche), 1:2000 in PBST, was performed o/n at 4°C. After several washes in PBST, embryos were washed with APT buffer (0.1 M Tris-HCl, pH 9.5, 0.15 M NaCl, and 0.1% Tween-20 in nuclease-free water) and with APTMg buffer (0.1 M Tris-HCl, pH 9.5, 0.15 M NaCl, 0.05 M MgCl_2_, and 0.1% Tween-20 in nuclease-free water) for 10 min. Finally, samples were incubated in nitro-tetrazolium blue-5 bromo-4-chloro-3′-indolyphosphate p-toluidine (Sigma-Aldrich), 0.033% in APTMg in the dark. Embryos were fixed for 1 h and mounted in 86% glycerol for microscopic observation. Images were captured using a Leica DM6 B Fully Automated Upright Microscope System.

### 2.11 Total RNA extraction, cDNA synthesis, amplifications, and sequencing

To quantify the transcription of *tiar*, *ttp*, and *g3bp*, total RNA was isolated from untreated and metal-treated embryos at mid-tailbud II and hatching larval stages using the NucleoSpin RNA XS kit (Macherey–Nagel, Düren, Germany). RNA concentration and purity were assessed using a Nanodrop ND-1000 spectrophotometer (Thermo Fisher Scientific), and RNA integrity was verified using electrophoresis. Reverse transcription from 1 μg of total RNA was performed with the ImPromII (Promega) kit. The obtained cDNA was used for qualitative and quantitative PCR according to the PCRBIO Classic Taq (PCRBIOSYSTEMS, London, UK) and HOT FIREPol EvaGreen qPCR Mix Plus (ROX) (Solis BioDyne) kit instructions, respectively. The melting profile was analyzed using qRT-PCR to verify the absence of genomic contamination. For each treatment, three pools of embryos from three different artificial fertilization events (biological triplicates) at the desired stages were analyzed by running each sample three times (technical triplicates).

Total RNA was extracted from *Ciona* adult intestines, and Cr-g3bp_PCRFw and Cr-g3bp_PCRRv primers (Supplementary Table S1) were used to amplify the retrotranscribed cDNA. Amplicons were separated using electrophoresis, and the corresponding bands were purified with the Wizard SV Gel and PCR Clean-Up System (Promega) kit, ligated in pGEM-T Easy Vector (Promega, Madison, WI, USA), and cloned in DH-5α *E. coli* cells. Plasmid DNA was extracted from positively screened colonies using the UltraPrep (AHN Biotechnologie GmbH) kit and sequenced by Eurofins Genomics to verify *g3bp* transcription in *C. robusta* (for *tiar* and *ttp* see Drago et al., 2021).

### 2.12 Data collection and statistical analyses

Relative values were obtained from qRT-PCR using the 2^−ΔΔCT^ (Pfaffl, 2001) mathematical model; the levels of transcription were normalized with respect to the housekeeping gene (*β-actin*; primer in Supplementary Table S1), to compensate for variations in the amounts of cDNA, and with respect to control (untreated) embryos, for each considered embryonic stage and metal-exposed embryos. Data are expressed as the mean of three biological samples (n = 3) ± standard deviation and were statistically compared using Duncan’s test (Snedecor and Cochran, 1980).

In the gene reporter assay, only completely electroporated embryos were used for confocal microscopy. The percentages of control and metal-treated embryos showing various signal locations ± standard deviations were compared with the χ^2^ test.

To validate the DNA cleavage achieved by the CRISPR/Cas9 sgRNAs, the relative proportion of DNA contained in each amplicon of the final gel was determined using ImageJ software, starting from the gel images. The cleavage efficiency was calculated according to the manufacturer’s instructions:

Cleavage efficiency = 1 – [(1 – fraction of cleaved bands) ½]; fraction of cleaved bands = sum of cleaved band intensities/(sum of cleaved and parental band intensities).

In gene knockout experiments, the area and length of trunk (in lateral view) and tail and the bending angle of the tail in mid-tailbud II embryos were measured according to Lu et al. (2020), using the ImageJ software. They were compared with the controls (specimens with *tiar*, *ttp* and *g3bp* normally expressed) with the Student’s *t* test. The same measures were not evaluated in hatching larvae as the phenotypes resulting from the knockout experiments were severely altered and, in most cases, it was not possible to distinguish clearly the trunk from the tail.

## 3 Results

### 3.1 Gene and protein organization is highly conserved across metazoans

The gene and protein organizations of C*. robusta* TIAR and TTP (Cr*-*TIARx1 and Cr-TTP, respectively) have been previously described (Drago et al., 2021). The Cr-g3bp_PCRFw and Cr-g3bp_PCRRv primers allowed us to amplify a region of 853 nt, the sequencing of which confirmed the putative *g3bp* sequence that we found in the ANISEED database (transcript ID: KY2019:KY.Chr1.2038.v1.SL1-1). This region, covering most of the CDS (Supplementary Figure S1), showed the highest similarity (100%) and identity (97.62%) to *Ciona intestinalis* G3BP2 (GenBank accession number: XM_002126499.5) in the NCBI database. The *in silico* reconstruction resulted in a whole transcript of 1797 nt that we named *cr-g3bp2* with 5′-UTR and 3′-UTR regions of 72 and 360 nt, respectively, and the CDS of 1365 nt, encoding a putative protein of 454 amino acids with a deduced molecular weight of 51.6 kDa (Supplementary Figure S1). The gene is located on chromosome 1, as revealed by the ghost database, and is organized into nine exons (length in nt from exon one to nine: 98, 82, 174, 103, 188, 174, 232, 113, and 201) and eight introns (length in nt from intron one to eight: 368, 226, 1700, 463, 418, 211, 308, and 229).

The multi-alignment analysis on G3BP2 amino acid sequences of tunicates (*Phallusia mammillata*, *Ciona savignyi*, *C. robusta*, *Halocynthia roretzi*, *Botrylloides leachii*), *Branchiostoma floridae, Latimeria chalumnae*, and *Patella vulgata*, the last three as representative of cephalochordates, vertebrates, and spiralian invertebrates, respectively, showed: i) the conserved N-terminal NTF2 domain (50.4% of identical amino acids among sequences), ii) the C-terminal RRM domain (33.8% of identical amino acids among sequences), iii), the central acid-proline-rich region and iv) the C-terminal arginine- and glycine-rich region (Supplementary Figure S2). In the RNP2 of the RRM domain, the valine (V) residue was present: it allows the classification of all analyzed amino acid sequences, including that of *C. robusta,* as G3BP2. In the sequence of *B. floridae*, which is the only G3BP sequence from cephalochordates found in NCBI, V is substituted with isoleucine (I), a derivative of G3BP1. Despite this, among the examined non-tunicate species, Cr-G3BP2 showed the highest identity (47.4%) and similarity (68%) with *B. floridae* (E-value 1.8e−40). Among all the species, the Cr-G3BP2 amino acid sequence showed the highest identity (78.4%) and similarity (90.1%) with that of *C. savignyi* (E-value 2.9e−107), as revealed by the LALIGN tool (Supplementary Table S2).

### 3.2 *tiarx1, ttp, and g3bp2* are expressed preferentially in mesendoderm and central nervous system

**FIGURE 1:**
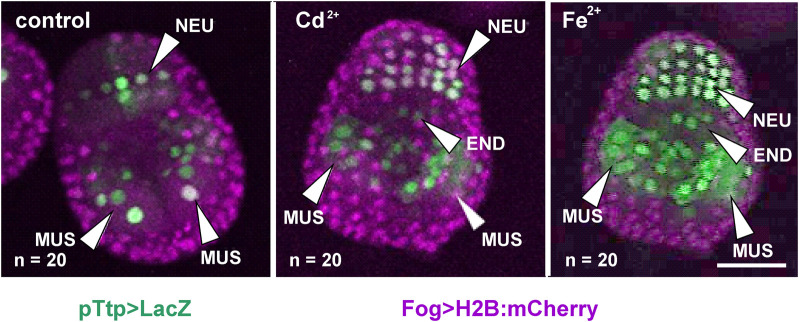
Gene reporter assay in mid-gastrula embryos, after electroporation of pTtp > LacZ (green), together with Fog > H2B:mCherry plasmid marking successful uptake of the electroporation mix (purple). Embryos are reported in control conditions, after cadmium (Cd^2+^) exposure and after iron (Fe^2+^) exposure. END: endoderm; MUS: muscle cells; NEU: neural plate. Scale bar: 50 μm.

The regulation of *tiarx1*, *ttp*, and *g3bp2* transcription was analyzed in mid-gastrula, mid-tailbud II, and hatching larva embryos of *C. robusta*. At the mid-gastrula stage, only the promoter of *cr*-*ttp* resulted active in the regions of the neural plate and muscle precursors (Figure 1). In metal-treated embryos, we could observe a significant (*p* < 0.001) increase of embryos showing an expansion of the region of expression which interested also the precursors of the endoderm in 60% of the embryos, as reported in Table 1.

**TABLE 1:** Activity of the promoter of *cr-ttp* in tissue precursors of mid-gastrula embryos, according to the gene report assay, under normal (control) and stress (exposure to Cd^2+^ and Fe^2+^) conditions. Frequencies of metal-treated and control embryos were compared with χ^2^ test.

Plasmid	Experimental conditions	Endoderm	Nervous system	Muscle cells
**Ttp > LacZ**	**control**	0/20 (0%)	20/20 (100%)	20/20 (100%)
	**Cd** ^**2+**^	12/20 (60%)***	20/20 (100%)	20/20 (100%)
	**Fe** ^**2+**^	12/20 (60%)***	20/20 (100%)	20/20 (100%)

In bold, statistically significant increments with respect to the related controls. ***: *p* < 0.001.

**FIGURE 2:**
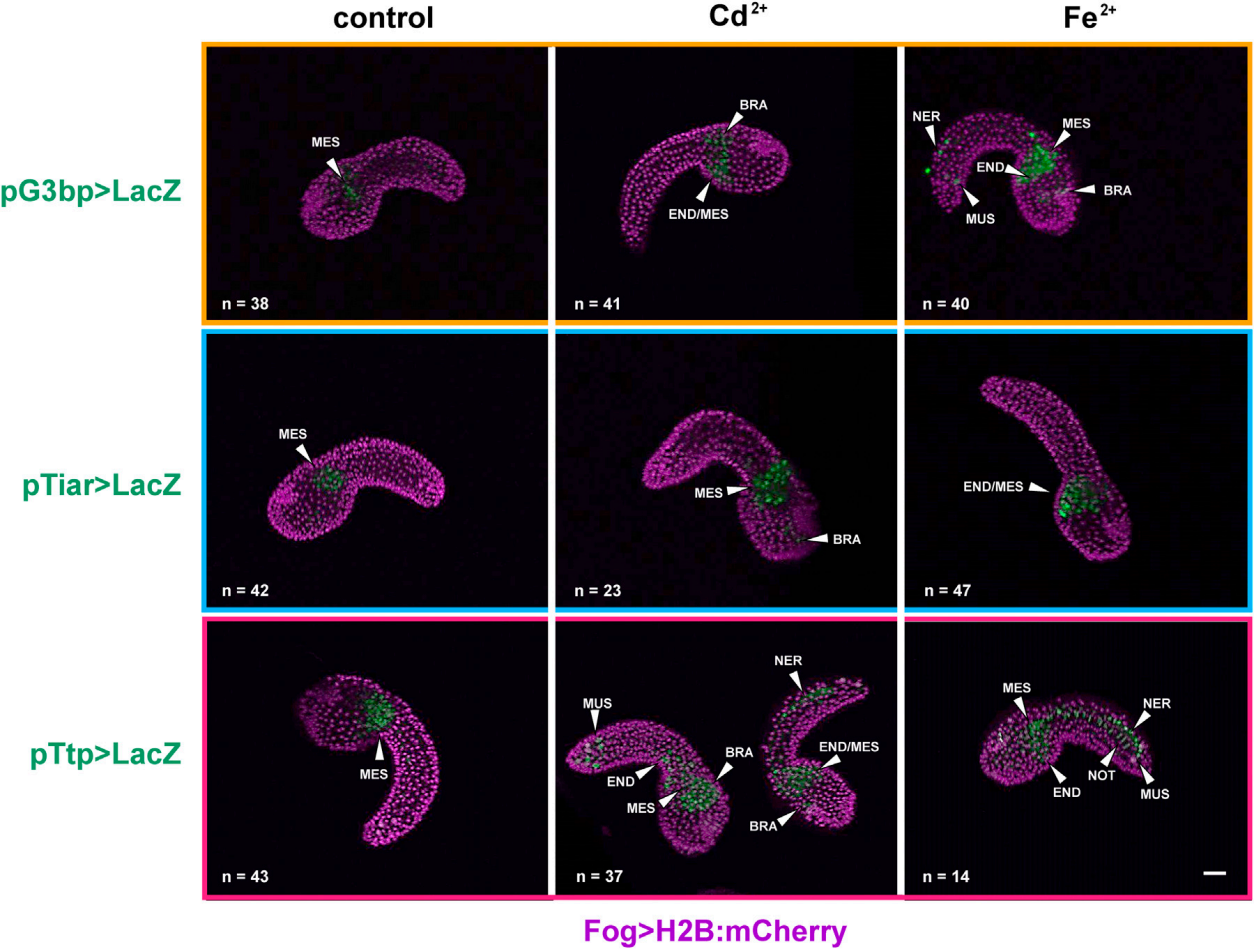
Gene reporter assay in mid-tailbud II embryos, after electroporation of pG3bp > LacZ (orange box), pTiar > LacZ (blue box), or pTtp > LacZ (red box) (green), together with Fog > H2B:mCherry plasmid marking successful uptake of the electroporation mix (purple). Embryos are reported in control conditions, after Cd^2+^ exposure and after Fe^2+^ exposure. MES: mesenchyme; END: endoderm; BRA: brain; NER: nerve cord; NOT: notochord; MUS: muscle cells. Scale bar: 100 μm.

In the mid-tailbud II stage, under all experimental conditions (control, Cd^2+^, and Fe^2+^), all the promoters were active in the mesenchyme of all the observed embryos. In the case of *cr-g3bp2*, after metal treatments, the activation was also visible in the endoderm, in the cells of the nervous system and in muscle cells of the tail in a certain fraction of the embryos. When embryos were exposed to Fe^2+^, a significant (*p* < 0.01) increase of the embryos with labelled nervous system cells was observed (Figure 2; Table 2).

**TABLE 2:** Activity of the promoters of *cr-g3bp2*, *cr-tiarx1* and *cr-ttp* in tissue precursors of mid-tailbud II embryos, according to the gene report assay, under normal (controls) and stress (exposure to Cd^2+^ and Fe^2+^) conditions. Frequencies of metal-treated and control embryos were compared with χ^2^ test.

Plasmid	Experimental conditions	Mesenchyme	Endoderm	Nervous system	Notochord	Muscle cells
**G3bp > LacZ**	**control**	38/38 (100%)	11/38 (29.0%)	14/38 (37%)	2/38 (5.3%)	7/38 (18.4%)
	**Cd** ^**2+**^	41/41 (100%)	12/41 (29.3%)	14/41 (34.1%)	1/41 (2.4%)	5/41 (12.2%)
	**Fe** ^**2+**^	40/40 (100%)	10/40 (25.0%)	23/40 (57.5%)**	0/40 (0%)	9/40 (22.5%)
**Tiar > LacZ**	**control**	42/42 (100%)	8/42 (19.0%)	9/42 (21.4%)	1/42 (2.4%)	4/42 (9.5%)
	**Cd** ^**2+**^	23/23 (100%)	4/23 (17.4%)**	13/23 (56.5%)***	1/23 (4.3%)**	1/23 (2.4%)**
	**Fe** ^**2+**^	47/47 (100%)	9/47 (19.1%)	18/47 (38.3%)**	1/47 (2.1%)	2/47 (4.2%)
**Ttp > LacZ**	**control**	43/43 (100%)	14/43 (32.6%)	23/43 (53.5%)	5/33 (11.6%)	11/43 (25.6%)
	**Cd** ^**2+**^	37/37 (100%)	9/37 (24.3%)	20/37 (54.0%)	2/37 (5.4%)	20/37 (54.0%)***
	**Fe** ^**2+**^	14/14 (100%)	5/14 (35.7%)***	8/14 (57.1%)***	1/14 (7.1%)**	7/14 (50.0%)***

In bold, statistically significant differences with respect to the related controls. **: *p* < 0.01; ***: *p* < 0.001.

As for *cr-tiarx1*, in addition to the mesenchyme, some embryos showed activity of the promoter in the trunk, brain and endoderm cells. After the exposure to Cd^2+^ and Fe^2+^, we could report a significant (*p* < 0.01) increase in the number of embryos in which the promoter was active in the brain (Figure 2; Table 2).

In the case of *cr-ttp,* the exposure to Fe^2+^ caused a statistically significant (*p* < 0.001) increment of embryos with the promoter active in the nervous system, endoderm and muscle cells; in few embryos, the promoter activation was also visible in the notochord. Cd^2+^ exposure led to a significant (*p* < 0.001) increase of embryos with the promoter active in the tail muscle cells (Figure 2; Table 2).

**FIGURE 3:**
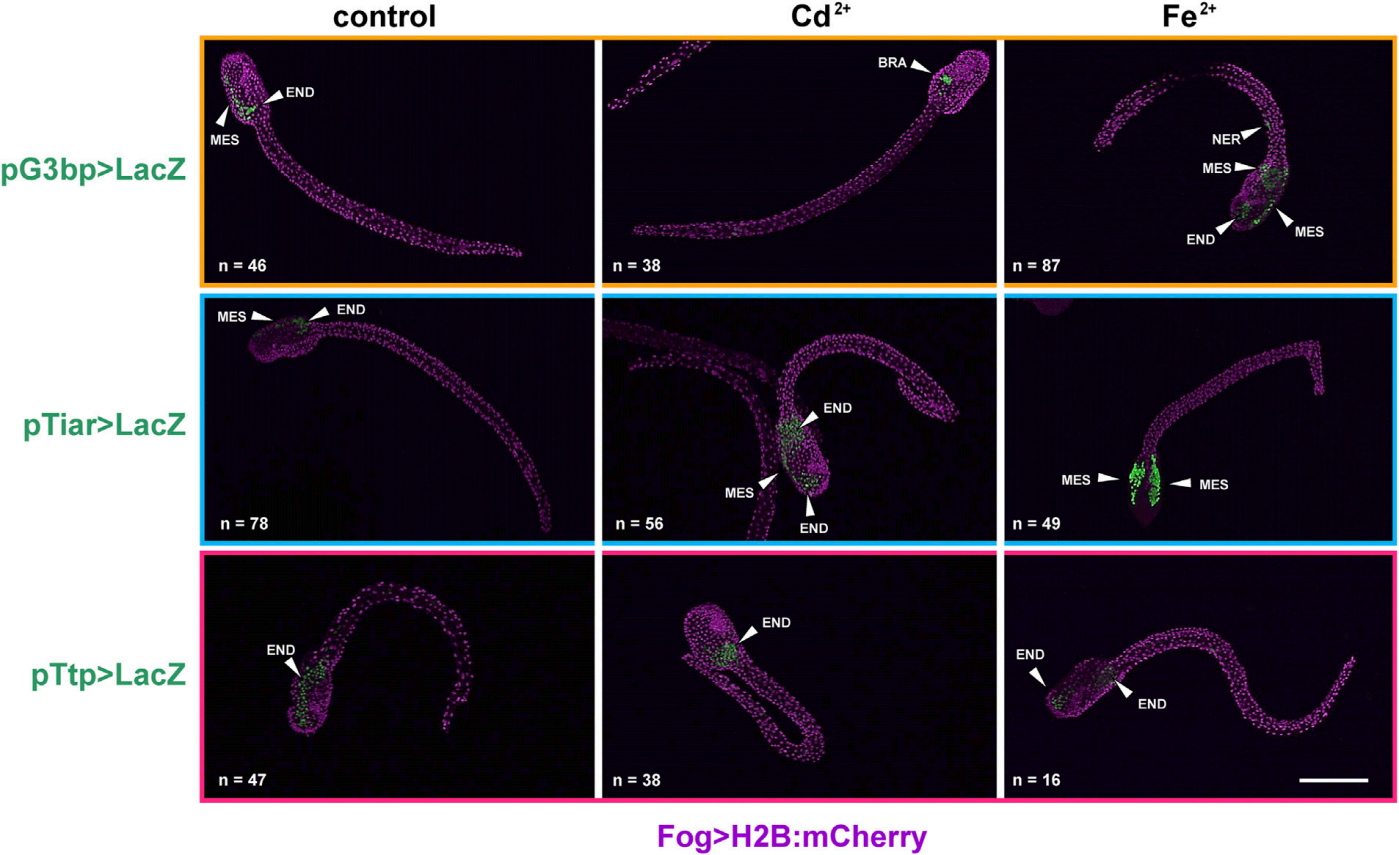
Gene reporter assay in hatching larvae, after electroporation of pG3bp > LacZ (orange box), pTiar > LacZ (blue box) or pTtp > LacZ (red box) (green), together with Fog > H2B:mCherry plasmid marking successful uptake of the electroporation mix (purple). Embryos are reported in control conditions, after Cd^2+^ exposure and after Fe^2+^ exposure. MES: mesenchyme; END: endoderm; BRA: brain; NER: nerve cord. Scale bar: 100 μm.

In hatching larvae, signs of activity for the considered genes in the tail were very rare. Conversely, in the trunk, *cr-tiarx1* and *cr-ttp* were particularly active in mesenchyme cells, for *cr-g3bp2* and *cr-tiarx1*, and endoderm cells, for *cr-ttp.* The anterior papillae were never labelled. In the Cd^2+^-treated specimens, the activation area increased in size in the case of *cr-tiar* and included the brain in the case of *cr-g3bp2*; no effects were visible for *cr-ttp*. When exposed to Fe^2+^, hatching larvae showed a general increase in the activation area for all the considered genes: it spreads in the trunk and, in the case of *cr-g3bp*, it included also the tail nerve cord After metal treatments, larvae with activation of *cr-tiarx1* and *cr-ttp* promoters in mesenchyme and endoderm cells increased significantly (*p* < 0.05). In the case of *cr-g3bp2*, a significant (*p* < 0.001) increase in the fraction of larvae with labelled endoderm cells was observed after Fe^2+^ exposure (Figure 3; Table 3).

**TABLE 3:** Activity of the promoters of *cr-g3bp2*, *cr-tiarx1* and *cr-ttp* in tissues of hatching larvae, according to the gene report assay, under normal (controls) and stress (exposure to Cd^2+^ and Fe^2+^) conditions. Frequencies of metal-treated and control embryos were compared with χ^2^ test.

Plasmid	Experimental conditions	Mesenchyme	Endoderm	Nervous system
**G3bp > LacZ**	**control**	18/46 (39.1%)	14/46 (30.4%)	14/46 (30.4%)
	**Cd** ^**2+**^	18/38 (47.4%)	16/38 (42.1%)	4/38 (10.5%)**
	**Fe** ^**2+**^	33/87 (37.9%)***	37/87 (42.5%)***	17/87 (19.5%)***
**Tiar > LacZ**	**control**	26/78 (33.3%)	30/78 (38.5%)	22/78 (28.2%)
	**Cd** ^**2+**^	25/56 (44.6%)**	22/56 (39.3%)*	9/56 (16.1%)**
	**Fe** ^**2+**^	24/49 (49.0%)***	19/49 (38.8%)*	6/49 (12.2%)**
**Ttp > LacZ**	**control**	15/47 (31.9%)	9/47 (19.2%)	14/47 (29.8%)
	**Cd** ^**2+**^	15/38 (39.5%)**	18/38 (47.4%)***	10/38 (26.3%)
	**Fe** ^**2+**^	8/16 (50.0%)*	6/16 (37.5%)***	2/16 (12.5%)***

In bold, statistically significant differences with respect to the related control. *: *p* < 0.05; **: *p* < 0.01; ***: *p* < 0.001.

### 3.3 Metals induce ectopic transcript enrichment

**FIGURE 4:**
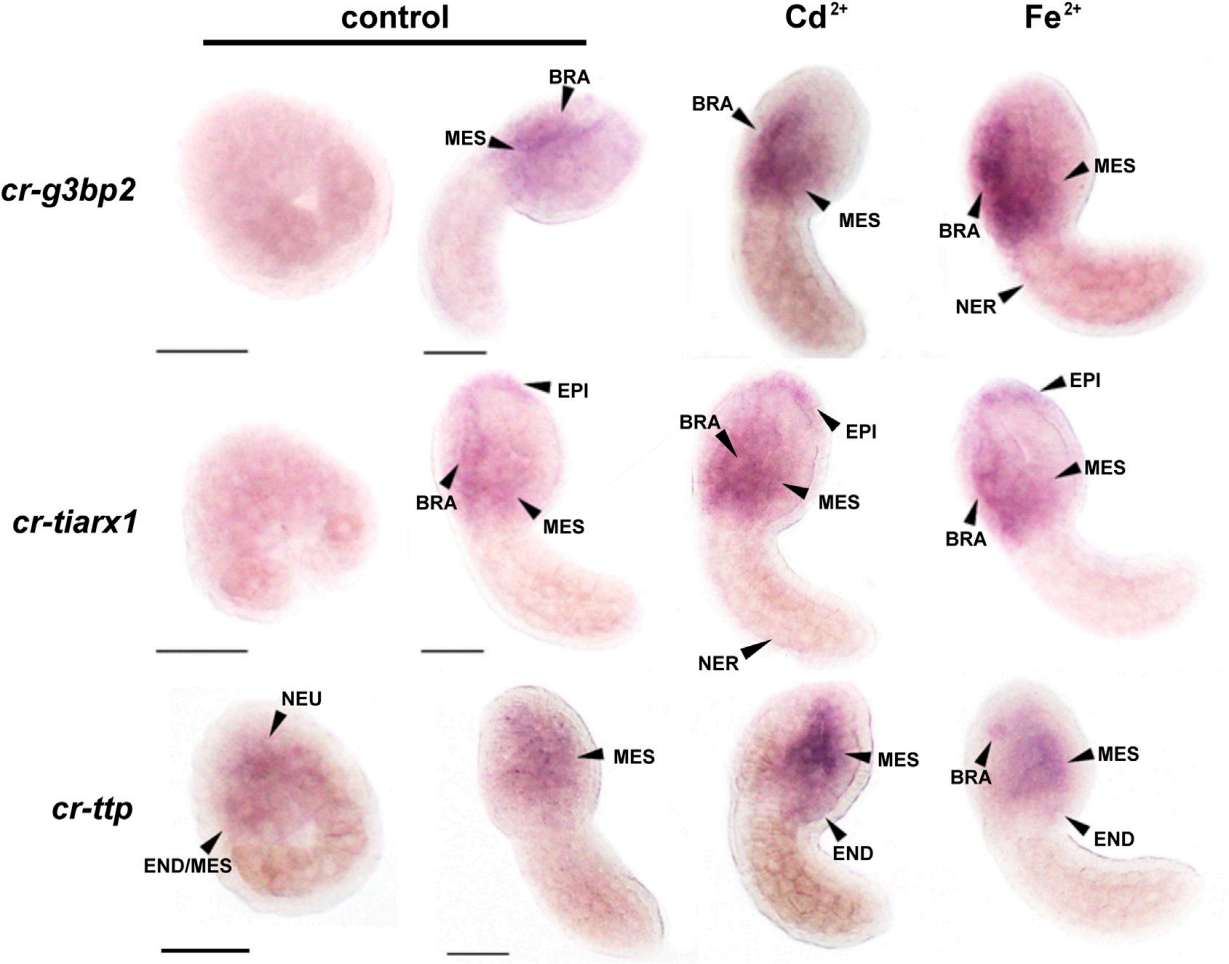
Whole mount ISH on mid-gastrula and mid-tailbud II embryos, with *cr-g3bp2, cr*-*tiarx1* and *cr-ttp* riboprobes, in control condition or in presence of Cd^2+^ or Fe^2+^. NEU: neural plate; END: endoderm; MES: mesenchyme; BRA: brain; EPI: epidermis; NER: nerve cord. Scale bar: 100 µm.

*cr-tiarx1*, *cr-ttp*, and *cr-g3bp2* transcription was tested at mid-gastrula and mid-tailbud II stages, in which the tunic had not yet formed and, therefore, the background signal related to its presence was not present. At the mid-gastrula stage, the only positive signal was observed for *cr-ttp*, which was consistent with the early activation of the reporter gene. The signal was specifically detected in the neural plate under all experimental conditions (Figure 4). *cr-g3bp2* and *cr-tiarx1* were not expressed at this stage (Figure 4). Conversely, all the studied genes were expressed at the mid-tailbud II stage in the mesenchyme and nervous system, including the nerve cord for *cr-g3bp2* and *cr-tiarx1*, after Fe^2+^ and Cd^2+^ exposure, respectively. The transcripts of *cr-tiarx1* were also present in the anterior epidermis, the region of origin of the larval papillae, important for adhesion. In addition, the endoderm of the trunk expressed *cr-ttp* at the mid-tailbud II stage (Figure 4). The absence of expression after the use of sense riboprobes was previously demonstrated (Drago et al., 2021).

### 3.4 *cr-g3bp2, cr-tiarx1* and *cr-ttp* mRNA expression increase after iron treatment

**FIGURE 5:**
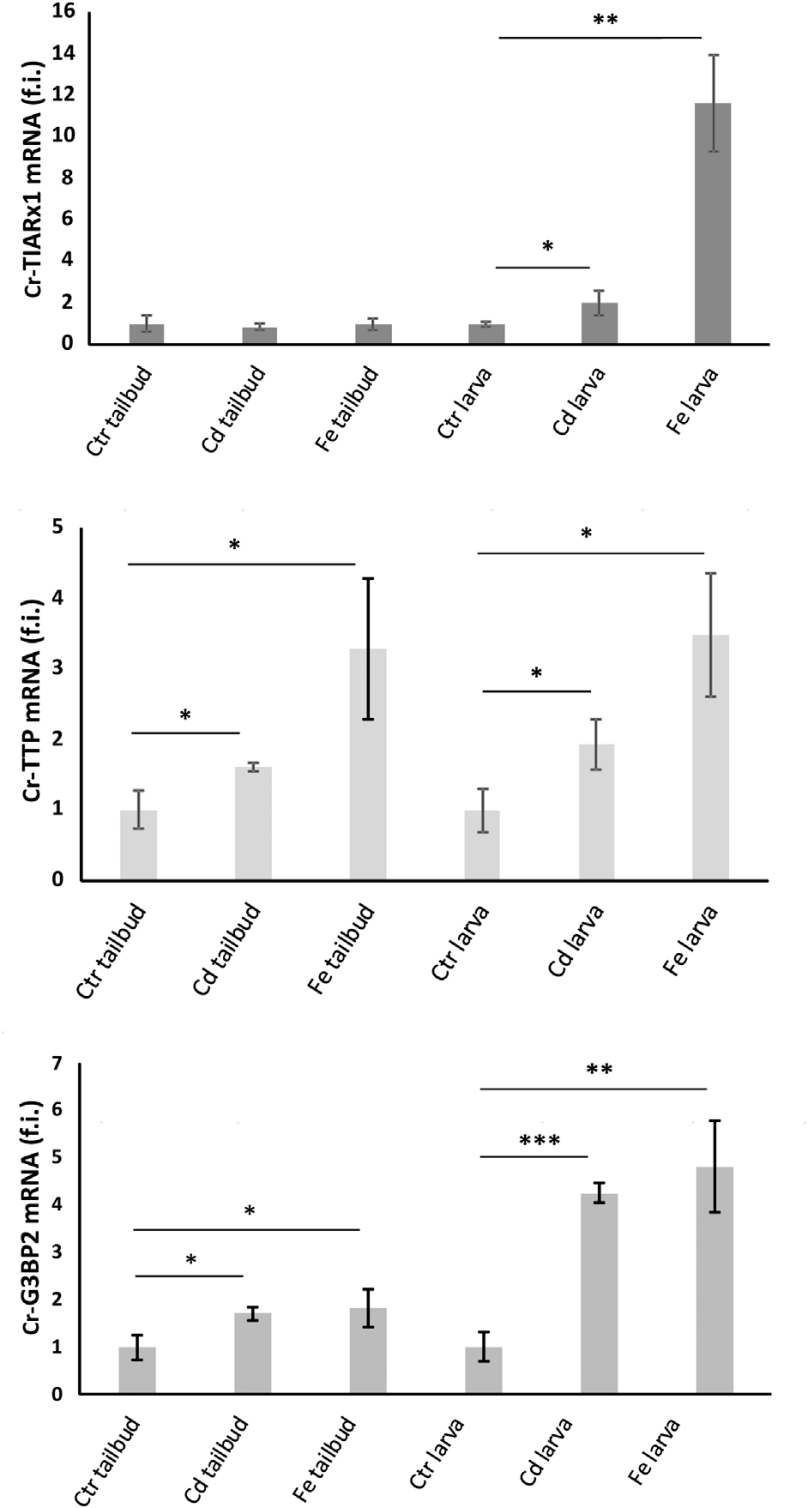
Relative expression levels (fold induction, f.i.) of Cr-TIARx1, Cr-TTP and Cr-G3BP2, in pooled mid-tailbud II embryos (tailbud) and hatching larvae (larva), developed in FSW without metal (Ctr) or with Cd^2+^ or Fe^2+^. Asterisks mark significant differences with respect to controls (***: *p* < 0.001; **: *p* < 0.01; *: *p* < 0.05).

*cr-g3bp2, cr-tiarx1* and *cr-ttp* gene expression was quantified in the mid-tailbud II and hatching larva embryos, both under control conditions and after metal exposure (Figure 5). The highest transcription levels were observed after Fe^2+^ exposure at the hatching larval stage for all studied genes. Except for *cr-tiarx1*, at the mid-tailbud II stage, a statistically significant increase (*p* < 0.05) in gene expression was always observed relative to the control group.

### 3.5 Knockout of SG genes greatly affects mesendodermal derivatives

Gel images resulting from Genomic Cleavage Detection assay, on genomic DNA extracted from initial tailbud embryos electroporated with Eef1a > Cas9 together with U6 > tiar_sgRNA3, U6 > ttp_sgRNA4, or U6 > g3bp_sgRNA2 vectors, are reported in Supplementary Figure S3. From the digestion reactions of the reannealed PCR amplicons, we were able to identify parental bands and cleavage products of the expected size. The cleavage efficiency for sgRNA2, targeting *cr-g3bp2,* was 20.3%; the value amounted to 23.8% for sgRNA3, targeting *cr-tiarx1*, and to 14.8% for sgRNA4, targeting *cr-ttp*. Controls obtained from genomic DNA extracted from embryos electroporated with Eef1a > Cas9 vector alone were also considered: in this case, no cleavage products were detected (data not shown). The gel image conditions were optimized using ImageJ software to reduce the background signal as much as possible. The third cleavage band detected in the case of *cr-tiarx1,* is due to naturally occurring single-nucleotide polymorphisms.

**FIGURE 6:**
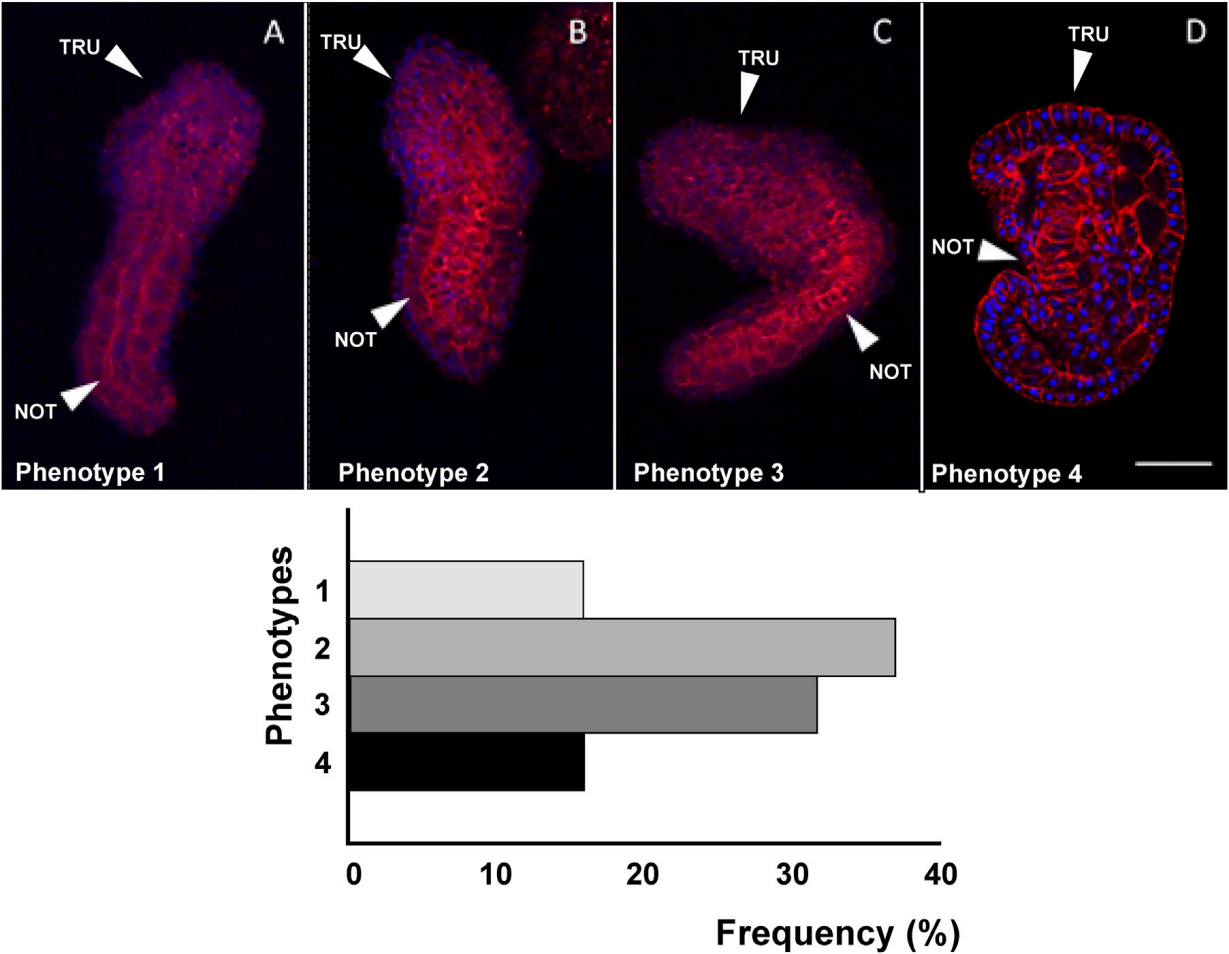
Four different phenotypes **(A–D)**, related to mid-tailbud II stage, resulting from *cr-g3bp2* knockout. Arrows indicate the notochord, as seen by Phalloidin staining (red) and nuclei labeled with DAPI/Hoechst (blue). Scale bar: 100 μm. The frequency (%) of phenotype is also reported. NOT: notochord; TRU: trunk.

**FIGURE 7:**
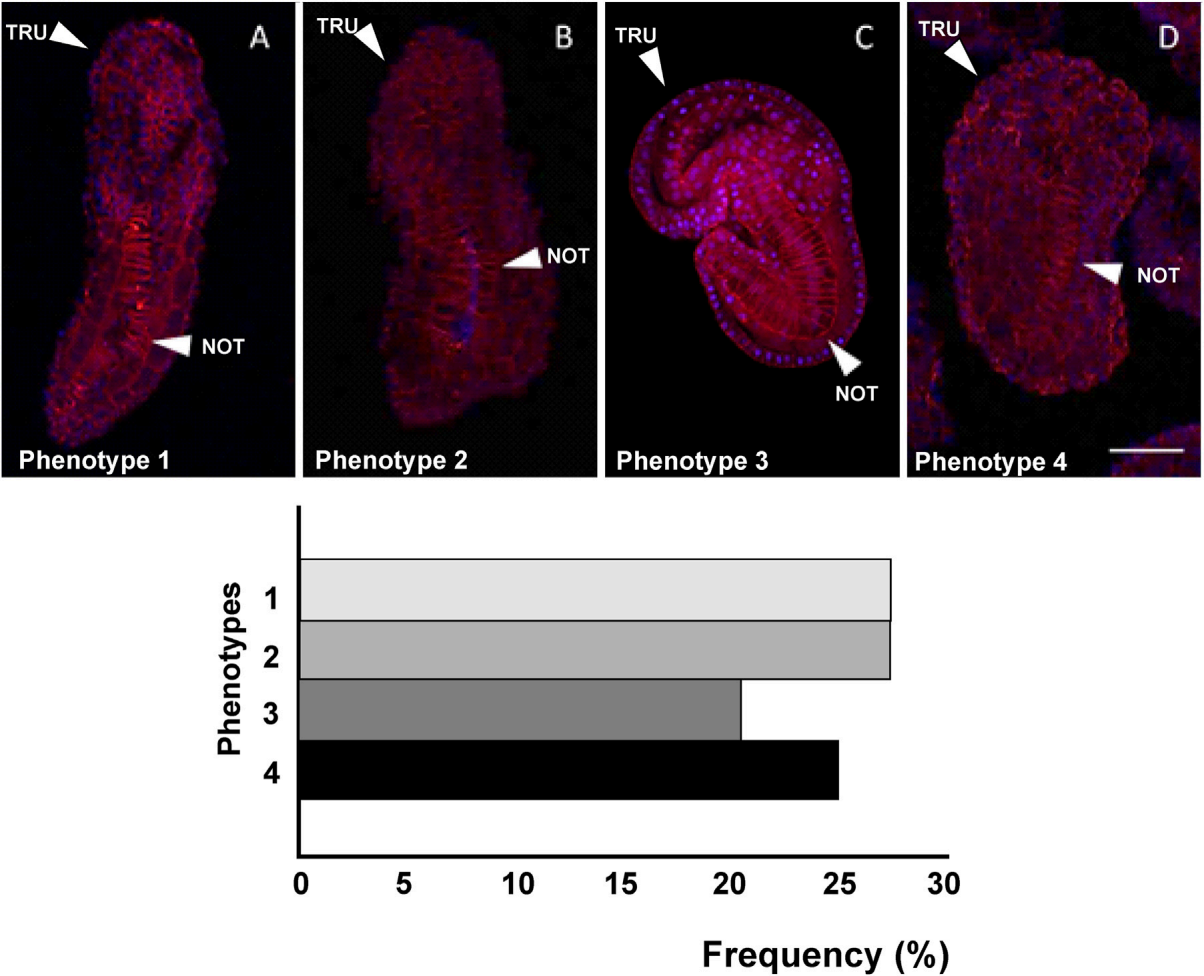
Four different phenotypes **(A–D)**, related to mid-tailbud II stage, resulting from *cr-tiarx1* knockout. Arrows indicate the tail and the notochord inside, as seen by Phalloidin staining (red) and nuclei labeled with DAPI/Hoechst (blue). Scale bar: 100 μm. The frequency (%) of phenotype is also reported. NOT: notochord; TRU: trunk.

**FIGURE 8:**
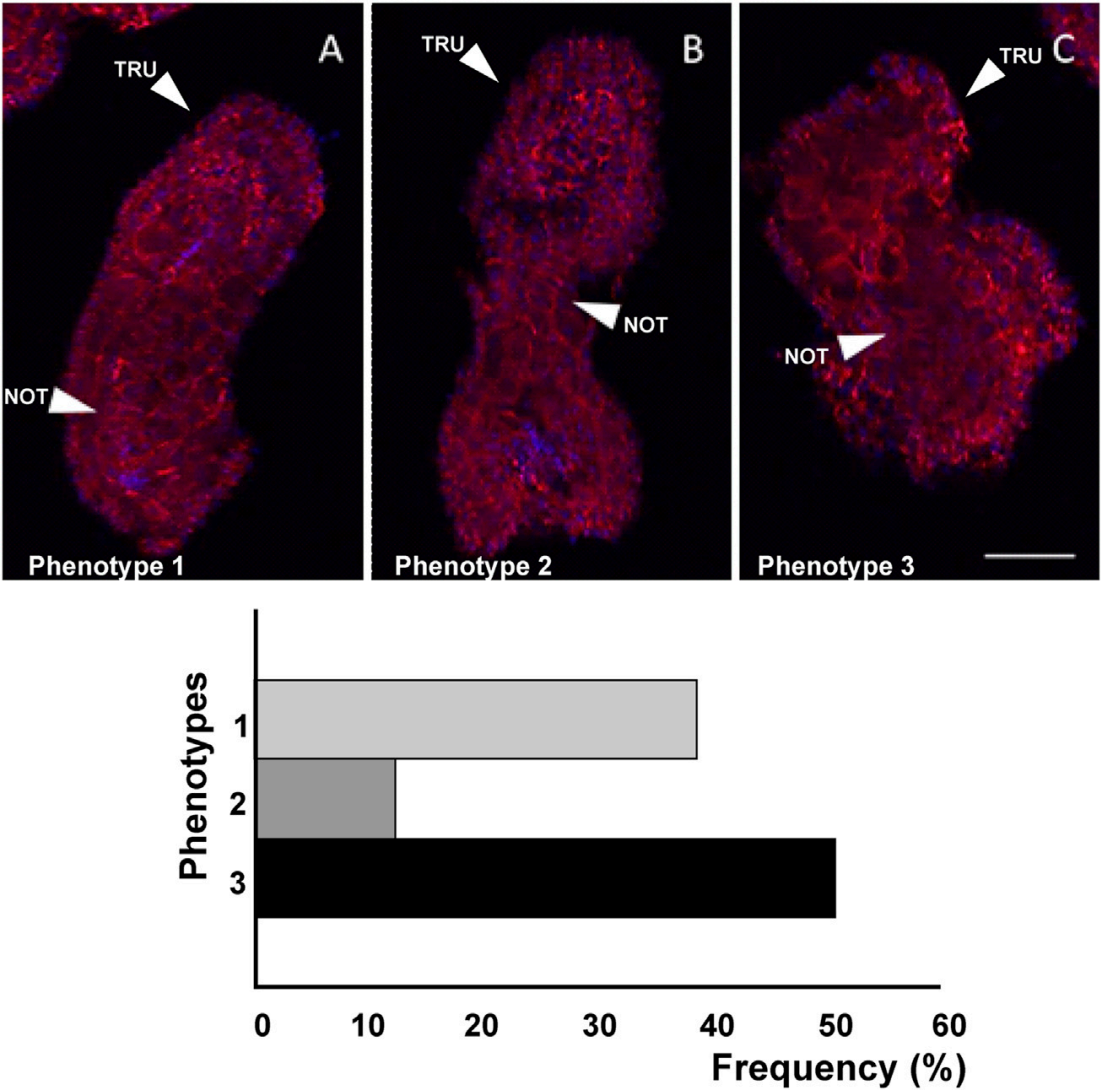
Three different phenotypes **(A–C)**, related to mid-tailbud II stage, resulting from *cr-ttp* knockout. Arrows indicate the tail and the notochord inside, as seen by Phalloidin staining (red) and nuclei labeled with DAPI/Hoechst (blue). Scale bar: 100 μm. The frequency (%) of phenotype is also reported. NOT: notochord; TRU: trunk.

**FIGURE 9:**
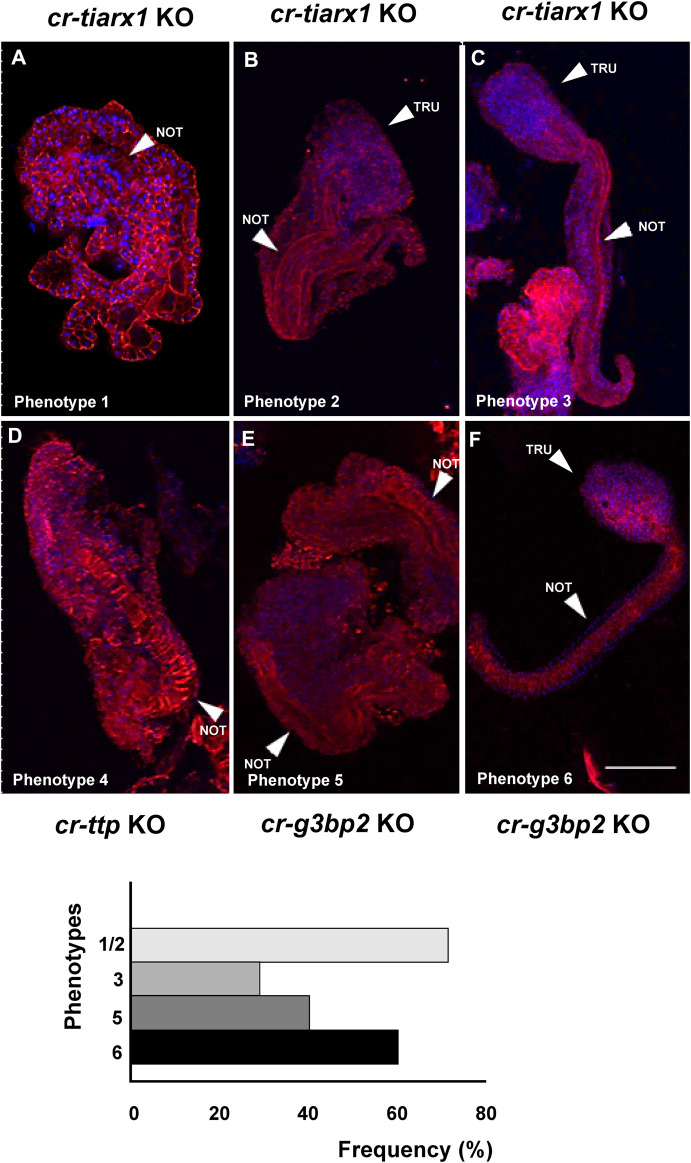
Six different phenotypes **(A–F)**, related to hatching larva stage, resulting from *cr-tiarx1* **(A,B,C)**, *cr-ttp* **(D)** or *cr-g3bp2* **(E,F)** knockout. Arrows indicate the tail and the notochord inside, as seen by Phalloidin staining (red) and nuclei labeled with DAPI/Hoechst (blue). Scale bar: 100 μm. The frequency (%) of phenotype is also reported. NOT: notochord; TRU: trunk.

The various phenotypes resulting from tissue-specific knockout of *cr*-*g3bp2, cr*-*tiarx1* and *cr*-*ttp*, using the sgRNAs reported above, can be found in Figures 6–8 for the mid-tailbud II stage, and in Figure 9 for hatching larva. The control phenotypes for the two analyzed stages are shown in Supplementary Figure S4.

As reported in Figure 6, in the case of *cr-g3bp2* knockout, we could distinguish four phenotypes: phenotype 1 (16%, n = 34), 2 (37%), 3 (32%) and 4 (15%). With respect to controls, the knockout of the gene caused a significant (*p* < 0.05) decrease in trunk and tail length in phenotypes 1–3 as well as a significant (*p* < 0.05) decrease in trunk area in phenotypes 2–4, and a significant (*p* < 0.001) decrease in tail area in all the observed phenotypes. In addition, we reported a significant (*p* < 0.05) increase in the bending angle of the tail in phenotypes 1, 3 and 4 and a significant (*p* < 0.01) reduction of the same angle in phenotype 3 (Table 4).

**TABLE 4:** Values of some parameters of mid-tailbud II developmental stage considered in comparing control with knockout specimens, expressed as mean ± SD.

Phenotypes	Trunk area (µm^2^)	Tail area (µm^2^)	Trunk lenght (µm)	Tail lenght (µm)	Tail Angle (°)
cr-g3bp2 knockout phenotypes					
Ctr	24,180.32 ± 2,845.35	24,550.3 ± 2,903.88	236.29 ± 21.57	330.67 ± 11.29	65.42 ± 4.63
1	13,731.09 ± 2,767.85	15,326.05 ± 2,236.90***	161.76 ± 19.86***	229.61 ± 10.59***	**72.4535** ± 2.72*
2	15,913.79 ± 1877.30***	15,747.46 ± 2,147.48***	154.53 ± 10.64***	240.67 ± 28.49***	41.69 ± 8.40
3	16,662.43 ± 5,482.24*	16,901.25 ± 1629.83***	161.40 ± 50.89**	237.08 ± 18.93***	60.32 ± 3.59**
4	17,390.13 ± 672.90**	16,437.95 ± 1885.15***	203.70 ± 95.78	318.38 ± 140.05	72.201 ± 4.79*
*cr-tiarx1* knockout phenotypes					
Ctr	24,180.32 ± 2,845.35	24,550.3 ± 2,903.88	236.29 ± 21.57	330.67 ± 11.29	65.42 ± 4.63
1	25,219.55 ± 1768.12	25,509.61 ± 1264.05	230.46 ± 7.47	290.19 ± 20.73**	42.18 ± 3.73***
2	20,623.60 ± 3,910.05	29,773.74 ± 4,780.96	153.80 ± 46.42**	248.55 ± 61.55*	34.58 ± 1.56***
3	22,748.73 ± 2,714.71	25,402.84 ± 9,242.72	156.63 ± 21.49***	246.99 ± 15.92***	83.83 ± 4.33***
4	25,405.19 ± 2084.83	27,422.90 ± 6,483.74	162.83 ± 11.17***	261.99 ± 35.69***	35.25 ± 4.67***
*cr-ttp* knockout phenotypes					
Ctr	24,180.32 ± 2,845.35	24,550.30 ± 2,903.88	236.29 ± 21.57	330.67 ± 11.29	65.42 ± 4.63
1	26,020.78 ± 3,318.03	29,142.11 ± 336.35**	227.97 ± 33.82	242.39 ± 36.94***	50.99 ± 2.71***
2	38,917.18 ± 1253.06***	33,262.44 ± 1549.44***	264.05 ± 32.97	298.54 ± 23.11*	42.73 ± 1.96***
3	27,367.60 ± 1818.66	23,618.17 ± 1618.42	171.17 ± 26.34**	378.49 ± 27.94***	68.66 ± 3.39

In bold, statistically significant differences with respect to controls. *: *p* < 0.05; **: *p* < 0.01; ***: *p* < 0.001.

Also after *cr-tiarx1* knockout, we could observe four phenotypes (Figure 7): phenotype 1 (27%, n = 44), 2 (27%), 3 (20%) 4 (25%). In this case, the knockout of the gene caused a significant (*p* < 0.01) reduction of the trunk length in phenotypes 2–4 and a significant (*p* < 0.05) decrease of the tail length in all the phenotypes and of the tail curvature in phenotypes 1, two and four and a significant (*p* < 0.001) increase in phenotype 3 (Table 4).

As a consequence of *cr-ttp* knockout, we distinguished three phenotypes (Figure 8): phenotype 1 (38%, n = 30), very similar to the phenotype 2 obtained after *cr-tiarx1* knockout, 2 (12%) with a bifurcated terminal tail and 3 (50%) which resulted highly malformed with unclear morphology of the trunk and tail regions. The trunk area was significantly (*p* < 0.001) increased in phenotype 2, whereas the tail area resulted significantly (*p* < 0.01) higher in phenotypes 1 and 2. Moreover, the trunk length was severely (*p* < 0.01) decreased in phenotype 3 and the tail length was significantly (*p* < 0.05) decreased in phenotypes 1 and 2 and significantly (*p* < 0.001) increased in phenotype 3. In phenotypes 1 and 2 a statistically significant (*p* < 0.001) decrease in tail angle was observed.

Regarding the hatching larval stage, specimens with a well-distinguished tail, even if larger than normal, were observed after *cr*-*tiarx1* and *cr*-*g3bp2* knockout in 29% (n = 59) and 60% (n = 40) of the observed larvae, respectively (Figures 9C, F). In all other cases, the embryos were highly malformed with apparent closure defects of the neural tube; the tail region was identified by the presence of a notochord where the tail was no so well distinguished from the trunk (Figures 9A, B, D, E). *cr*-*ttp* knockout resulted in 100% of the embryos (n = 40) with a single malformed phenotype (Figure 9D) with unclear trunk-tail border and the tail region marked by the presence of the notochord whose cells do not seem to undergo tubulogenesis.

## 4 Discussion

Homeostasis is essential for ensuring the survival of organisms and, consequently, the perpetuation of species. This is especially true for organisms living under variable environmental conditions such as lagoons, marinas, or harbors. Recent studies have confirmed the ability of ascidians (sessile species of tunicates, a sister group of vertebrates) to cope with stressful conditions by activating antioxidant (Franchi et al., 2012; Ferro et al., 2018) and immune responses (Parrinello et al., 2015; Franchi and Ballarin, 2017; Peronato et al., 2020; Marino et al., 2023). These responses are mainly detectable in hemocytes, representing the detoxification system of ascidians (Franchi et al., 2011; 2014).

SGs are ribonucleoproteic cytoplasmic foci that control and modulate global protein synthesis (Drago et al., 2021; Drago et al., 2023). G3BP, TIAR, and TTP are three molecular components of SGs that have been extensively studied in vertebrates. Aggregation of TIAR and G3BP is fundamental for SG formation, as it leads to temporary AU-rich mRNA silencing, thus ensuring normal cell proliferation and differentiation. An increasing number of reports indicate that SGs exert their action not only in the presence of stressful conditions but also when cells require proper control of protein synthesis. Because early development and differentiation require strict control of cell activity, it is conceivable that SGs play a role in these processes. The role of SGs in embryogenesis remains poorly understood, although some data from studies on mice (Ramos et al., 2004; Zekri et al., 2005; Kharraz et al., 2010; Sánchez-Jiménez and Izquierdo, 2013; Liu et al., 2023) and *C. elegans* (Huelgas-Morales et al., 2016) suggest their involvement in the above processes.

In the present study, we aimed to increase our knowledge of the role of *cr*-*g3bp2, cr*-*tiarx1* and *cr*-*ttp*, the orthologues of mammalian *g3bp, tiar* and *ttp*, in the early development of a chordate invertebrate, the solitary ascidian *C. robusta*, under control conditions and after metal-induced stress conditions.

After electroporation of the vectors with promoters for *cr-g3bp2, cr-tiarx1* and *cr-ttp* we were able to visualize the regions in which the regulation of gene expression occurred. In the mid-tailbud II stage, the mesenchyme was the main tissue involved under all experimental conditions, whereas under stress conditions caused by metal (Fe^2+^ and Cd^2+^) exposure, the expression of the studied gene was ectopically activated especially in cells of the nervous system and the endoderm.

Mesenchymal cells located in the trunk will give rise to hemolymph cells and body-wall muscles of adults (Passamaneck and Di Gregorio, 2005). Our previous studies confirmed that the orthologues of *tiarx1* and *ttp* are expressed in the circulating immunocytes of the solitary ascidian *C. robusta* (Drago et al., 2021) and the colonial species *B. schlosseri* and *B. primigenus;* in these last two species, hemocytes also transcribe *g3bp2* (Drago et al., 2023; 2024). Circulating ascidian immunocytes control both phagocytosis and inflammation, which are the two typical cellular responses of innate immunity. We previously demonstrated that, in *B. schlosseri*, microinjection of a specific anti-TIAR antibody in the colonial circulation significantly reduced the ability of phagocytes to ingest foreign target cells and of cytotoxic cells to degranulate and trigger the inflammatory response (Drago et al., 2023). In *C. robusta*, transcripts for TIARx1 and TTP are mainly present in granulocytes, an immunocyte type that can act as a detoxifying organ capable of accumulating metal ions and migrating to the tunic, as observed in *B. schlosseri*, where they undergo apoptosis and clearance by phagocytes (Franchi et al., 2014; Drago et al., 2021; 2023). The ISH results showed that *cr-g3bp2* is also transcribed in immunocytes, strongly suggesting its involvement in the immune response (Supplementary Figure S5).

Endodermal cells present in the trunk and tail (endodermal strands) will give rise to endostyle, branchial sac, and digestive organs of adults (Hirano and Nishida, 2000). Our previous studies confirmed *tiarx1* transcription and relative protein expression in the endostyle, stomach, and intestine of *B. schlosseri* (Drago et al., 2023). Using qRT-PCR, in *C. robusta*, *cr-tiarx1* and *cr-ttp* transcription upon metal exposure (Cu^2+^, Zn^2+^, and Cd^2+^) was confirmed in the intestine, which is one of the first organs to contact xenobiotics in filter feeders (Drago et al., 2021). *cr-g3bp2* is also expressed in the intestine of *Ciona* adults, with a maximum transcriptional activity at 24 and 72 h and a minimum at 48 h of exposure to Cd (Supplementary Figure S6). This trend was already observed for the other two genes and is likely related to the post-transcriptional control operated by SGs; once perceived the acute stress at 48 h, cells disassembled their SGs to unlock the translation of mRNAs for anti-stress proteins.

In the mid-tailbud II stage, the transcription of all studied genes was also observed in the tail (endodermal strand, nerve cord, notochord, and muscle cells), which was not confirmed in later development. Hatching larvae will undergo metamorphosis, during which the tail is resorbed and does not contribute to the formation of the adult body, which is probably the reason for the absence of transcription of *g3bp2, tiarx1* and *ttp* in this developmental stage. Conversely, the activation of the studied genes in the hatching larval stage was visible in the endoderm and mesenchyme cells, especially in embryos exposed to Fe^2+^.

The central nervous system of ascidian larvae includes a visceral ganglion and a sensory vesicle in the trunk, connected by a neck, and caudal nerve cord (Olivo et al., 2021). Our data, indicating the activity of *cr-ttp* promoter in the neural plates of mid-gastrula embryos, suggest the involvement of TTP in the development of the ascidian brain. In addition, the reported transcriptional activities of *g3bp2*, *tiar*, and *ttp* in the mammalian brain, where they are implicated in both signal transduction and RNA metabolism (Kennedy et al., 2001; Rayman and Kandel, 2017; Li et al., 2019), fit our observation indicating the importance of the three genes for a correct brain development. The activity of the promoter in brain precursors is increased by metal exposure in both mid-gastrula and mid-tailbud II embryos, suggesting a decisive role of these genes in controlling cell homeostasis and facing stressful conditions.

Our whole-mount ISH results confirmed that the transcription of the studied genes occurs mainly in the brain, mesenchyme, and endoderm of mid-tailbud II embryos, indicating the possible role of *cr-g3bp2, cr-tiarx1* and *cr-ttp*, in immune responses and defense of the adult digestive system, in addition to development. We also quantified the transcriptional activity of the three genes in mid-tailbud II embryos and hatching larvae using qRT-PCR in untreated and metal-exposed specimens. Except for *cr-tiarx1* at the mid-tailbud II stage, all studied genes showed a statistically significant increase in gene transcription in metal-exposed conditions with respect to the controls at both developmental stages, reaching the highest level after Fe^2+^ exposure. According to our previous hypothesis on post-transcriptional control operated by SGs formed by G3BP2, TIARx1 and TTP, acute stress should lead to the unlocking of mRNAs from disassembled SGs to codify proteins to face adverse conditions with the consequent decline in transcription of the related genes. A possible explanation for the observed increase in expression during metal-induced stress conditions is that cells need to codify the key proteins for the assembly of SGs to store AU-rich mRNAs for proteins that are active in the control of cell proliferation and differentiation and cellular metabolism, rather than proteins active in attenuating stress conditions. AU-rich mRNAs for anti-stress proteins, such as metallothionein and glutathione (Franchi et al., 2011; 2012), could conversely be free in the cytoplasm and promptly translated. In stressed embryos, SGs could act in controlling the mRNAs for TNF, p53, STAT5B, and MYC, valuable for ensuring normal embryogenesis (Piecyk et al., 2000; Johnson and Blackwell, 2002; Jackson et al., 2006; Marderosian et al., 2006; Liao et al., 2007; Vignudelli et al., 2010; Geng et al., 2015; Takayama et al., 2018; Zhang et al., 2021). It is worth to note that, as evidenced by ISH, *tiarx1* mRNA is present in the anterior epidermis of the mid-tailbud II embryos from which the larval anterior papillae will derive. However, no expression can be found in the same region of embryos and larvae subjected to gene reporter assay. We can argue that the presence of mRNA for TIARx1 in the epidermis, and, presumably, of the corresponding protein, triggers a negative feedback which induces the block of the promoter activity.

The importance of *cr-g3bp2, cr-tiarx1* and *cr-ttp* in controlling cell proliferation and differentiation is highlighted by our CRISPR/Cas9 experiments. In the absence of stress, the knockout of the above genes affected normal embryogenesis, leading to malformations at the level of the tail, as evidenced by the unnatural curvatures, and the trunk, sometimes showing a decrease in size, mainly in the case of *g3bp2* knockout. This can be due to either modified patterns of cell proliferation or altered spacing among the cells. We are currently trying to solve this aspect of the altered phenotypes. In addition, the trunk and the tail were often undistinguished from each other, especially in hatching larvae. In the case of *cr-g3bp2*, the gene knockout seemed to cause an arrest in trunk development at the late tailbud III stage, which was confirmed by the few studies in the literature, according to which the inhibition of G3BP causes growth retardation or embryonic lethality due to an increase in neuronal cell death by apoptosis (Zekri et al., 2005). The knockout of *cr-tiarx1* and *cr-ttp* leads, among others, also to defects in the notochord which seems to assume an unnatural location suffer from intercalation defects and incapability to undergo tubulogenesis.

## 5 Conclusion

This is the first study of SG molecular markers in ascidian embryogenesis. Phenotypes resulting from the transfected embryos of *C. robusta* revealed that *cr-g3bp2, cr-tiarx1* and *cr-ttp* are required to ensure normal embryogenesis, which is probably supported by the formation of SGs during stress conditions to allow the control of mRNA translation in proteins regulating cell proliferation, differentiation, and metabolism. Future studies will be directed to investigate the extent of the synthesis of the above-reported proteins during *C. robusta* development.

## Scope statement

Tunicates occupy the key phylogenetic position of vertebrate sister group: this justifies the increasing interested towards these marine invertebrates which can contribute to a better understanding of biological processes difficult to study in vertebrates. Ascidians are sessile tunicates present in all the seas and oceans. In previous studies, we demonstrated the presence of *tiar, ttp*, and *g3bp*, three genes involved in stress granules formation, in both solitary and colonial ascidians. In the colonial species *B*. *schlosseri* and *B. primigenus*, we also demonstrated their involvement in non-embryonic development. With the present study, we investigated the transcriptional activity of the above genes during embryogenesis of the solitary ascidian *Ciona robusta* under both physiological and stress conditions. Using CRISPR/Cas9, we evaluated the effects of the gene knockout on embryonic development. In addition, we used gene reporter assay, whole-mount *in situ* hybridization and quantitative real time PCR to study time and tissue specificity and to quantify gene transcription. Results can help us to add new information on the evolution of stress granules in chordates.

## Data Availability

The original contributions presented in the study are included in the article and Supplementary Material. Sequences of cr-g3bp2, cr-tiarx1 and cr-ttp have been deposited in GenBank with the following access numbers: PP997437, PP997438 and PP997439, for cr-g3bp2, cr-tiarx1 and cr-ttp, respectively.

## Abbreviations

ASW, artificial seawater; CDS, coding region; CRISPR, clustered regularly interspaced short palindromic repeats; EtOH, ethanol; FSW, filtered sea water; G3BP, ras-GTPase-activating protein SH3-domain-binding protein; ISH, *in situ* hybridization; NTF2, N-terminal nuclear transport factor 2; o/n, overnight; p-bodies, processing bodies; PBS, phosphate-buffered saline; PBST, PBS with Tween-20; qRT-PCR, quantitative real-time PCR; RNP, ribonucleoprotein; RRM, RNA-Recognition Motif; SG, stress granules; sgRNA, single guide RNA; SSC, sodium citrate solution; STAT5, signal transducer and activator of transcription 5b; TIAR, TIA 1 related nucleolysin; TNF-α, tumor necrosis factor-alpha; TTP, tristetraprolin; UTR, untranslated region.
