# Supplementary material for: Stress granule-related genes during embryogenesis of an invertebrate chordate
Source: Front Cell Dev Biol. 2024 Aug 1;12:1414759. doi: 10.3389/fcell.2024.1414759 (PMC11324471; doi:10.3389/fcell.2024.1414759)
Supplement: Supplementary file 1 [file DataSheet1.PDF]

Supplementary Material

Stress Granule-Related Genes During Embryogenesis of an Invertebrate Chordate

Laura Drago[1], Alessandro Pennati[2], Ute Rothbächer[2], Ryuji Ashita[3], Seika Hashimoto[3], Ryota Saito[3], Shigeki Fujiwara[3], Loriano Ballarin[1,*]

*** Correspondence:** Loriano Ballarin: loriano.ballarin@unipd.it

1 Supplementary Figures and Tables

1.1 Supplementary Figures

Supplementary Figure 1. cDNA sequence of Cr-G3BP2 and deduced amino acid sequence. 5'- and 3'-UTR regions are underlined. In grey the sequence obtained with amplicon sequencing.

```
   1   cta tgt tta tta act ttt aaa ttt ctg act tgt gcg taa tat ttc tat ttc ctt cca cct
  61   att act caa gaa atg gtt atg atg gca aaa cca agt cca att caa gtc gga cgt gaa ttt
                        M   V   M   M   A   K   P   S   P   I   Q   V   G   R   E   F
 121   gta cga caa tat tac acc ctg tta aac aaa gca cca gaa tta tta tac aga ttc tac agt
        V   R   Q   Y   Y   T   L   L   N   K   A   P   E   L   L   Y   R   F   Y   S
 181   atg cac tca tcc tat gtc cat gga ggc aga tac tgt aat ggg gaa cca gaa aag cca gtc
        M   H   S   S   Y   V   H   G   G   R   Y   C   N   G   E   P   E   K   P   V
 241   att ggt caa aat gaa atc cac acc aaa att gac tcg ctt gag ttc cga gat tgc cac acc
        I   G   Q   N   E   I   H   T   K   I   D   S   L   E   F   R   D   C   H   T
 301   aag att cga caa gtc gat gcc cat tct acc atc gga agc ggg att gtt gtc cag gtt act
        K   I   R   Q   V   D   A   H   S   T   I   G   S   G   I   V   V   Q   V   T
 361   gga gaa ctt tcc aac agt gga atg cct ctg cgt cga ttc atg caa acg ttc gtg ctt gct
      G   E   L   S   N   S   G   M   P   L   R   R   F   M   Q   T   F   V   L   A
 421   cct cag ggg gac aat cct tac aag ttt tat gtc cac aat gat att ttc cgt tac caa gat
      P   Q   G   D   N   P   Y   K   F   Y   V   H   N   D   I   F   R   Y   Q   D
 481   gaa gtg ttt cat gat gat caa caa act gat cga aca gat gac gaa aca gaa gaa gag agt
        E   V   F   H   D   D   Q   Q   T   D   R   T   D   D   E   T   E   E   E   S
 541   gag gcc atc gct tca gtc acg gcg ttc cag gac act tat tat aac caa acc aac aat gag
        E   A   I   A   S   V   T   A   F   Q   D   T   Y   Y   N   Q   T   N   N   E
 601   gaa aat gtc aat gga ttg gag caa cag gtg aag aac atg aaa gtt gaa tca ccc gag atc
        E   N   V   N   G   L   E   Q   Q   V   K   N   M   K   V   E   S   P   E   I
 661   gaa caa cca gtt att gaa cct tca cca act cct tca cct gtt cct gat gaa aga gag cct
      E   Q   P   V   I   E   P   S   P   T   P   S   P   V   P   D   E   R   E   P
 721   acc cca ccc cct acc acc aat aca ctt gac cca tcc ccc gaa ccc cct caa gaa gag att
      T   P   P   P   T   T   N   T   L   D   P   S   P   E   P   P   Q   E   E   I
 781   tcc gaa ccc cca ccc cca tcc aaa ccc ttc tct tgg gca gac ctt gca tca aag aac acc
        S   E   P   P   P   P   S   K   P   F   S   W   A   D   L   A   S   K   N   T
 841   cca gca cga agc aac aca cag caa gga aca gtt gtt aaa gcc cca cca aag cct gag cca
      P   A   R   S   N   T   Q   Q   G   T   V   V   K   A   P   P   K   P   E   P
 901   gtt gaa ccc gct gag tca gca cca aaa cct ccg cgt cag cct cgg cag aat caa cgc ttc
      V   E   P   A   E   S   A   P   K   P   P   R   Q   P   R   Q   N   Q   R   F
 961   act gca ccc aag gaa gaa gaa cga gcg tat ggt gat cga aac gat gca aga aga ccc aga
        T   A   P   K   E   E   E   R   A   Y   G   D   R   N   D   A   R   R   P   R
1021   gac gca cca agt ggt gtg gtt cgt tac cct gac aac cag cag atc ttc gtt gga aat tta
      D   A   P   S   G   V   V   R   Y   P   D   N   Q   Q   I   F   V   G   N   L
1081   cca att gac atc aag gag gca gat tta aaa aat cat ttt gca gaa ttt gga aat gta ctt
      P   I   D   I   K   E   A   D   L   K   N   H   F   A   E   F   G   N   V   L
1141   gaa gtg aga atc aac cac tca cat tct aac aac cca agc ttt gga ttt gtc atc ttt gag
      E   V   R   I   N   H   S   H   S   N   N   P   S   F   G   F   V   I   F   E
1201   agt cct agt gct gtt gaa aaa gtg ctg gag att atg ccg aca caa tac aaa aac aac cag
```

```
                  S   P   S   A   V   E   K   V   L   E   I   M   P   T   Q   Y   K   N   N   Q
1261               cgc att aac att gag gaa aaa aag caa cgt aat gcg agg gat gca cgt cgt ggt ggg gat
                  R   I   N   I   E   E   K   K   Q   R   N   A   R   D   A   R   R   G   G   D
1321               cca cgc aga ggt gct ggg gac aac cgt gct cgt cgt ggt ggt ggt gcg cct cag atg agg
                  P   R   R   G   A   G   D   N   R   A   R   R   G   G   G   A   P   Q   M   R
1381               cgg gac aga caa ggg tct cga gac gat ggt cgc aat tac aat gca aat cga cga taa tat
                     R   D   R   Q   G   S   R   D   D   G   R   N   Y   N   A   N   R   R   -
1441               tca gca att tta cta ttt tat aca agc gag ttc tcg ttg cat ggt ata aca gct gaa taa
1501               gaa gtt atc tct taa gtt cat tca ttt cag gct tgc tca gta ctc cct ttc aca ctg tac
1561               cac gct ttt cct tcc aca atg gat gta act atc gta ttt att caa gac cca cct ttt acc
1621               aag ctg taa atc cga ttc aag ctt gta aaa tgt caa aaa caa cga tag gaa aat gtt aaa
1681               aac gtt aat aaa gta tcg cac taa cat tta tgt aaa caa ttc gat tac tgt taa aga aag
1741             atc tcc aat gag tgt ctg ttg ttc ttt tga gtc cta ata aag gca ata ata tta att
```

Supplementary Figure 2. Schematic G3BP2 domain organization. The RRM domain, with RNP2 and RNP1 motifs boxed, respectively, with continuous and a dotted line, is highlighted in blue, whereas the NTF2 domain is highlighted in yellow. In RNP2 motif, the valine residue (V) which allows to discriminate G3BP2 from G3BP1 is in white and bold. The central acid- and proline-rich region shows the glutamic acid (E) and aspartic acid (D) residues, defining the acid-rich region, in black and bold, whereas the proline (P) residues, defining the proline-rich, in red and bold. Closely spaced arginine-glycine repeats (RGG, RGP, GRG, GGG), in C-terminal region, are in green and bold. *= identical amino acids (completely preserved), := very similar amino acids (semi-conservative substitution), .= similar amino acids (conservative substitution); numerals refer to character counts.

```
Phallusia mammillata      ----------------MVMMEKPSPIQVGREFVRQYYTLLNKAPELLHRFYSINSSYVHG  44
Ciona savignyi            ----------------MVMMEKPSPIQVGREFVRQYYTLLNKAPELLYRFYSMNSSYVHG  44
Ciona robusta             ----------------MVMMAKPSPIQVGREFVRQYYTLLNKAPELLYRFYSMHSSYVHG  44
Halocynthia roretzi       ----------------MVRMENPSPIQVGREFVRQYYTLLNKAPDMLHRFYGVNSSYVHG  44
Botrylloides leachii      ----------------MVRMNHPSPFQVGREFVRQYYSLLNKAPEILHRFYGVNSSYVHG  44
Latimeria chalumnae       -----------------MVMEKPSPLLVGREFVRQYYTLLNKAPDFLHRFYGRNSSYVHG  43
Patella vulgata           -----------------MVMETPSPQCVGREFVRQYYTLLHEAPRHLHRFYSHNSCFVHG  43
Branchiostoma floridae    MVMVMTTDSPQSTVTRDVRELHVSSQCVGREFVRQYYTLLNQAPEHLHRFYSHNSSFLHA  60
                                           :     *   **********:**::**  *:***. :*.::*.

Phallusia mammillata      GR-YCNGEPEQPVIGQNEIHLKINSLKFHDCRTKIRQVDAHSTIGSGVVVQVTGELSNEG  103
Ciona savignyi            GR-YSNGEPEKPVIGQTEIHTKIDSLDFHDCHTKIRQVDAHSTIGSGIVVQVTGELSNSG  103
Ciona robusta             GR-YCNGEPEKPVIGQNEIHTKIDSLEFRDCHTKIRQVDAHSTIGSGIVVQVTGELSNSG  103
Halocynthia roretzi       SSTFRSGETEEAVIGQMAIHKKITALQFKECHTKVRQVDAYSTIGSGVVVQVIGELSNNR  104
Botrylloides leachii      IG-YCHGESDEEVIGQTAIHKKIMSLHFKECHTKVYQVDAHSTLNDAVVVQVIGELSNNR  103
Latimeria chalumnae       GL-DASGKPAEAVYGQAEIHKKVMSLQFSECHTKIRHVDAHATLNDGVVVQVMGELSNNG  102
Patella vulgata           GV-EKPGEELPPVTGQSEIHKKIMSLNFRDCHAKIRQVDSQSTVGEAVVVQVTGELSNNG  102
Branchiostoma floridae    SC-DFGEHVEDPVIGQQDIHKKIMSLNFRDCHAKIRQVDSHPTLGNGVVVQVTGELSNNG  119
                                 .    * **  ** *: :*.* :*::*: :**:  *:...:**** *****.

Phallusia mammillata      MPLRRFMQTFVLAPQGDNPYKFYVHNDIFRYQDEVFMDNVPNDRSDGEQESEEEL-EQSQ  162
Ciona savignyi            LPLRRFMQTFVLAPQGDNPYKFYVHNDIFRYQDEVFLDESSTERVEEEQAVGESD--EEA  161
Ciona robusta             MPLRRFMQTFVLAPQGDNPYKFYVHNDIFRYQDEVFHDDQQTDRTDDETEE------ESE  157
Halocynthia roretzi       NPLRRFMQTFVLAPQGDNPYKFYVHNDVFRYEDEVFQEDVPQDRLEEDQASEELE-EEDP  163
Botrylloides leachii      KPLRRFMQTFVLAPQGDNPYKFYVHNDIFRYEDEVYRDDMPAERQNEEEHISDHEVDDDP  163
Latimeria chalumnae       QPMRKFMQTFVLAPEGSVPNKFYVHNDIFRYEDEVFGDSEGELDEESD---EEVE--EEP  157
Patella vulgata           QQMRRFMQTFVLAPQ--SPKKYYVHNDIFRYQDEVFHDNDSDVDITEE---CDVE--SEV  155
Branchiostoma floridae    EPMRRFMQTFVLAPQ--SPKKYYVHNDIFRYQDEVFEDSDYEAQERAGESEGEIE--AEP  175
                            :*:*********:    * *:*****:***:***: :.                  .

Phallusia mammillata      APTQ--VPAYQDKYSSQPATNQNLDEPTTLQQPPVMTNGLDQRLEESPVNE--------P  212
Ciona savignyi            QAPV--VPAYQDAYLDQQANHD------------LAMNGVVQQMETINVEA--------P  199
Ciona robusta             AIAS--VTAFQDTYYNQT-NNE------------ENVNGLEQQVKNMKVES--------P  194
Halocynthia roretzi       QTQT--VPQF-DNHSGYYQTQA----------AQIISNGIEQRDDAQETQLEPRQPSPSP  210
Botrylloides leachii      EPTQ--MPSHCENHMN--AQNQ----------IQQIGNKIEQHMDNQAANS-DSQPSPSP  208
Latimeria chalumnae       EERQPSPEPVQENTSSTYYE------------SHPVTNGLE------------------  186
```

```
Patella vulgata             ENGQQ-VEPQGDPS-NFYANT-----------DSPISNGTAHVQERIE------------  190
Branchiostoma floridae      EPTKP-PAPEQSPLEPAVFEP-----------QQTVSNGDIHLEESPPEVVP--------  215
                                       .                         *

Phallusia mammillata        VSNIPDVQEDQ------------------TPSPTRSLTPEIKREPTPTPAIEDLQLD---  251
Ciona savignyi              EIEQPEI----------------------EESPAQSPQPEEEREPTPPPTTTTIDPT---  234
Ciona robusta               EIEQPVI----------------------EPSPTPSPVPD-EREPTPPPTTNTLDPS---  228
Halocynthia roretzi         VTSAPPSPAAGTVQPPDSHCDQELMAPAST-SDTEPETN-IESDMFPPSATEDSQPDTVS  268
Botrylloides leachii        --PSPPIVNDA-----------QVFEPIQDPTPMEPEGP-EES-AFTLS--NDSQ-----  246
Latimeria chalumnae         ----EPLEE---------------------------PAPEPEPEVEPEPKHEELKPEVEE  215
Patella vulgata             ---SPPLKE---------------------------PEMKE--EEDI-----------EE  207
Branchiostoma floridae      --PQPPVEE---------------------------PAPPPEQEETP-----------EP  235


Phallusia mammillata        --EPPKV-----VEQP-KEEEAIVETKPTAPKILSWADRASKNTPAQSKTQ----QGTVV  299
Ciona savignyi              --PPPV-------------EEEVIETPAPSKPFSWADLASKNTPARSNVQ----QGTVV  274
Ciona robusta               --PEPP-------------QEEISEPPPPSKPFSWADLASKNTPARSNTQ----QGTVV  268
Halocynthia roretzi         STDPPLPAAGTTSTSS-AVTSVLPAEPEPTSQAFSWAALASKNTPQQHNIQ----QGTVV  323
Botrylloides leachii        --EPKIDSD------P-IDTKDDLSEKDESSQVTSWAALASKNTPAQHNFQ----QGTVV  293
Latimeria chalumnae         KALEELEE---KAPSPTPVEPPS--TPQEPPKAYSWASVTSKNLPPCGT-TSSGIPPHVV  269
Patella vulgata             DKY----E---EEPVPKSPEPPVEEEPPAEPKPFSWAALASKKTPGGNIPPASQVNMAAN  260
Branchiostoma floridae      EPYEPQQE---TAPEVKEPEPPK--EQQPEPKKFSWAALASKNTTASPQATTTGLPPTVI  290
                                                           :  ***  :**:               .

Phallusia mammillata        KVH-QQPEPTEPTE-APPRAQRPQRPNQRNYQRDDDRDRGFGDRSDQPRRSGPIGAGTTG  357
Ciona savignyi              KVVQPEP---EPVE-AAPKPARQPRANQR-FNAPKEEDRSYGDRNDARRQRE--------  321
Ciona robusta               KAP-PKPEPVEPAE-SAPKPPRQPRQNQR-FTAPKEEERAYGDRNDARRPRD--------  317
Halocynthia roretzi         KVNQQN-EPANLGE-SQPPRAQRSARGPVRTGYREDNDRGSGDRG---------------  366
Botrylloides leachii        KMNQAH-EVGNVDQ-AQKPAQRPPRSHPSQ---PPPPRDDSGDAG---------------  333
Latimeria chalumnae         KAPA-----SQPRVESKPETQTQSPRVREQRPRERPGMPVRGP---------RLGREGGE  315
Patella vulgata             KPQ-TTSTESKSDQNEAPQPQRQARPVPRDRNREREKTSSF-S--------R--ADGDGD  308
Branchiostoma floridae      KSQQVPPKPAEPRDAGAPQPQRAPRPQREGQDFRRPRPGGPGR--------REMGEGEEG  342
                            *         :

Phallusia mammillata        PPGGRDQMPIRYPDQQQIFVGNLPYDISESDLKDHFADFGKVIEVRINHSH--SNNPSFG  415
Ciona savignyi              APGGGGGGSVRYPDNQQIFVGNLPYDIKEVDLKNHFTDFGNVIEVRINHSH--SNNPSFG  379
Ciona robusta               APS----GVVRYPDNQQIFVGNLPIDIKEADLKNHFAEFGNVLEVRINHSH--SNNPSFG  371
Halocynthia roretzi         -GDGNQRRSIRYPDNQQIFVGNLPYEITESDLREHFTSYGDILEIRINHSH--TSNPSFG  423
Botrylloides leachii        -GDMGVRRGTRYPDNQQIFVGNLPYEITENDLREHFAEFGEIIDVRINHSH--TSNPSFG  390
Latimeria chalumnae         QNESDGRRIVRYPDSHQLFVGNLPHDIDESELKEFFMSFGNVVELRINTKGVGGKLPNFG  375
Patella vulgata             NDSLSGRRMPRYPDSHQLFVGNLPHNILEKDLKSFFEHYGNVMELRINTKSGGGKVPNFG  368
Branchiostoma floridae      RPETRVQRPSRYPDSHQLFIGNLPHDINEDELKDHFAHYGNVMELRINTKSGGGRVPNFG  402
                                      ****.:*:*:**** :* * :*:..*  :*.::::*** .      *.**

Phallusia mammillata        FVIFEECQAVDRVLEVMPTHYKDKHRINIEEKKQRSARDNNR-RIGGDQRRGPGMMGDGR  474
Ciona savignyi              FVIFENPSSVEKVLEIMPTQYKNNQRINIEEKKQRNARDP---RRGGDSRRGAGDIRN--  434
Ciona robusta               FVIFESPSAVEKVLEIMPTQYKNNQRINIEEKKQRNARDA---RRGGDPRRGAGDNRA--  426
Halocynthia roretzi         FIIFQEPKSVEEVLAQMPTTFKNNKRINIEEKKQRAPMR------GEGGRRGGGGGDMRR  477
Botrylloides leachii        FVIFEDPKSVEEVLRQMPTTFKNNKRINIEEKKQRGLN--------RDGRRGPGGWDARR  442
Latimeria chalumnae         FVVFDDSDPVQRILSAKPIMFRGEVRLNVEEKKTRAAREREI--RGGDDRRDIR-----R  428
Patella vulgata             FVVFDSPDPVQQILSNKPIRYNGDHRLNVEEKKPRGDGNRPSSGRGGMSRGGMNSGGSGR  428
Branchiostoma floridae      FVVFDDPEPVQKILDSKPIMFRGEHRLNVEEKKARGEGRGRGDSRGPP----------R  451
                            *::*:. . *:.:*   *  :... *:*:**** *

Phallusia mammillata        GARRGGGGGPMG----IR--RDRQGS-----REARDGQPRPS------HNTGQR---R    512
Ciona savignyi              ----RRGGGSTQ----MR--RDRPGS-----RDDARNYSSNR--------------R    462
Ciona robusta               ----RRGGGAPQ----MR--RDRQGS-----RDDGRNYNANR--------------R    454
Halocynthia roretzi         DN---ARGNRMQQRAPPR--RDRGGS-----REGQGSREGP----------GSR---K    512
Botrylloides leachii        DN---NRGGRMQNRGPPR--KEWGGP-----KEGQRPNNYG----------PRR----    476
Latimeria chalumnae         NDRGPGG-PRILGSGMMRDREGRGPPPRGGMAQKSGPLSGRGMGQGEGRFTGRR----    481
Patella vulgata             GGFGGGR-GGMNRGGFGNRSEGRGGNSN--VGGGRGGNSGGGGGGGGGFSNARR----    479
Branchiostoma floridae      GDRGPPR-GPG-------APAGRGG-GP--SGGGRGPPPPKGGGAGGGPPRDRYPPRR    498
```

Supplementary Figure 3. Genomic Cleavage Detection Assay gel analyzed with ImageJ software. Lane profiles for *cr-tiarx1, cr-ttp* and *cr-g3bp2*, referring respectively to sgRNAs 3, 4 and 2, are reported. In lanes "+" are visible the cleavage products, in lanes "-" are visible the negative controls (no detection enzyme used from the kit). P: parental band; 1: first cleavage product; 2: second cleavage product; M= molecular weight. Cleavage efficiencies (%) are also reported.

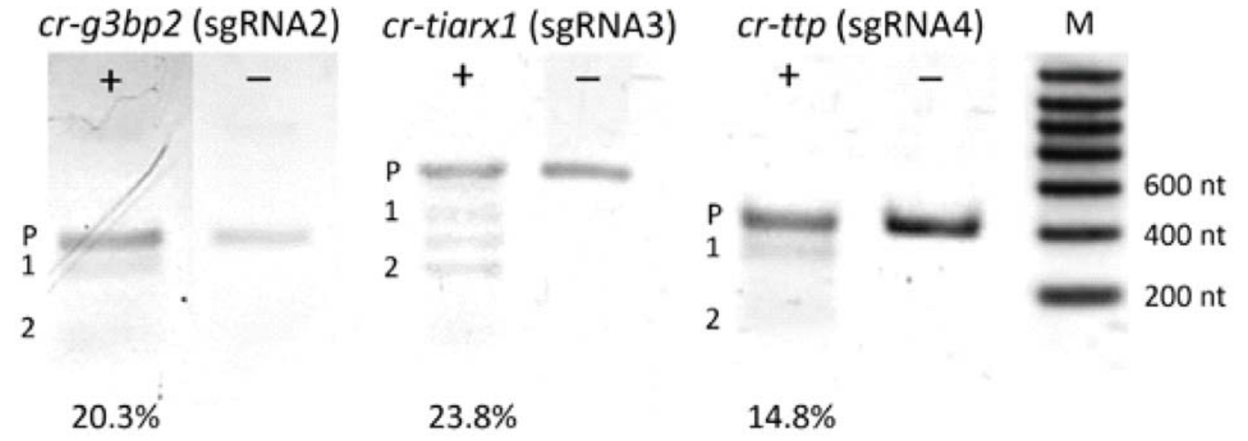

Supplementary Figure 4. Control phenotypes resulting from electroporation of FoxD>Cas9 only, for mid tailbud II (A) and hatching larva (B) stages. Arrows indicate the tail and the notochord inside, as seen by Phalloidin staining (red) and nuclei labeled with DAPI/Hoechst (blue). Scale bar: 100 µm.

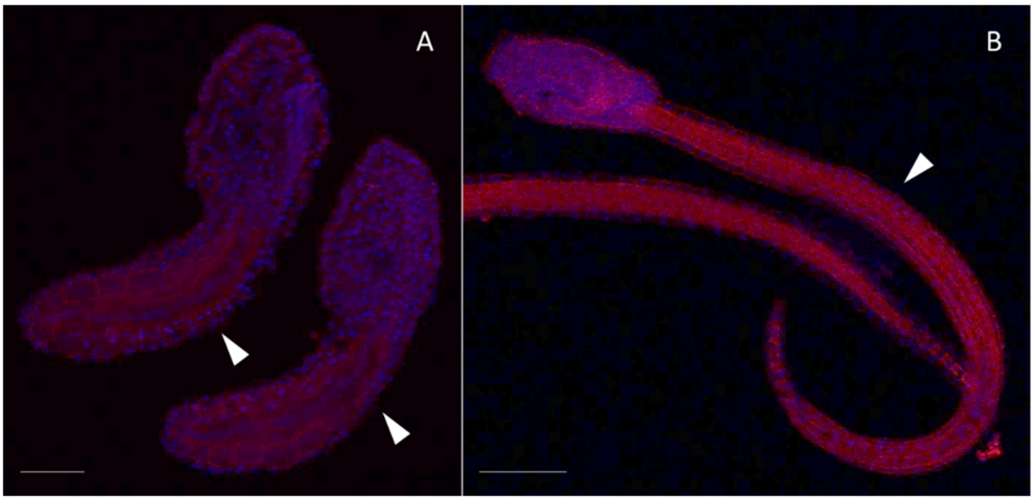

Supplementary Figure 5. ISH with anti-sense (A, B) and sense (C) *Cr-g3bp2* biotinylated riboprobe, on hemocytes collected from 72 h Cd (10 µM)-treated adults. Mc: morula cell; Ug: unilocular granulocyte. Scale bar: 10 µm.

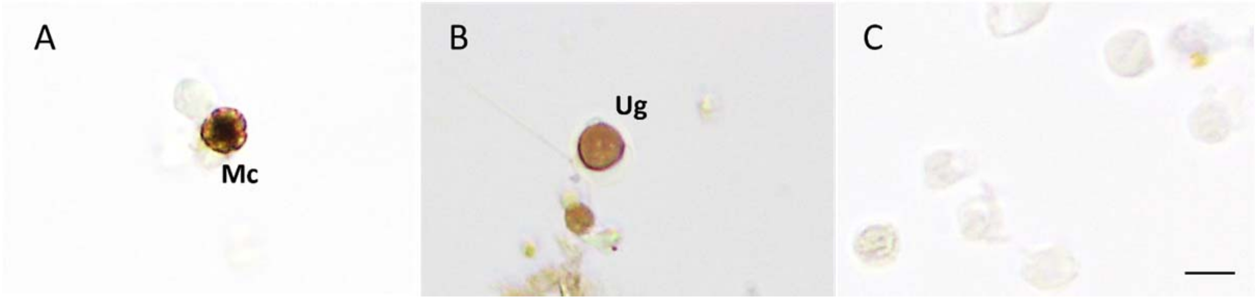

Supplementary Figure 6. Relative transcription levels (fold induction, f.i.) of Cr-G3BP2, in intestine from adults (n= 3) treated with Cd (10 µM). Data are normalized with respect to controls. Asterisks mark significant differences with respect to controls (***: $p < 0.001$).

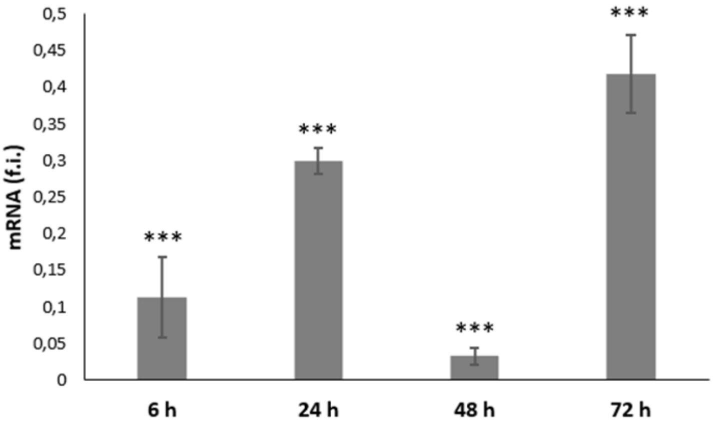

1.2 Supplementary Tables

Supplementary Table 1. Oligonucleotide sequences, and relative melting temperatures (Tm), used in gene reporter assay (highlighted in blue), CRISPR/Cas9 (highlighted in yellow) and qRT-PCR (highlighted in green). Bold residues represent vector overlapping sequences.

**Primers**	**Sequences 5'-3'**	**Tm (°C)**
Cr-g3bp_genereporterFw	**GACACTATAGAACTCGAG**ATGTGCCATTAAAGACCATTATTAA	73
Cr-g3bp_genereporterRv	**GGAGCAGTCATTTTT**TTCTTGACTAATAGGTGGAGGGG	75
Cr-tiar_genereporterFw	**GACACTATAGAACTCGAG**CGAGTACGTGCAACAAGAAATCC	75
Cr-tiar_genereporterRv	**GGAGCAGTCATTTTT**GACAAAACTTACAGTAGCAAAATTCAGAAAAC	74
Cr-ttp_genereporterFw	**GACACTATAGAACTCGAG**GCTCTCTCGTTTTAAGTGTGGAG	73
Cr-ttp_genereporterRv	**GGAGCAGTCATTTTT**GGCATTAATATTATTAAATACTTCAGTCCTACC	72
LacZ_IF_Fw	**AAAAATGACTGCTCC**AAAGAAGAAGCGTAAG	71
LacZ_IF_Rev	**CTCGAGTTCTATAGTGTC**ACCTAAATCGTATG	67
Cr-g3bp_gRNA1Fw	**AGATGG**CACTCATCCTATGTCCATGG	61
Cr-g3bp_gRNA1Rv	**AAAC**CCATGGACATAGGATGAGTG**CC**	61
Cr-g3bp_gRNA2Fw	**AGATGG**GTTTGCATGAATCGACGCAG	61
Cr-g3bp_gRNA2Rv	**AAAC**CTGCGTCGATTCATGCAAAC**CC**	61
Cr-g3bp_gRNA3Fw	**AGATGG**AGACCCAGAGACGCACCAAG	64
Cr-g3bp_gRNA3Rv	**AAAC**CTTGGTGCGTCTCTGGGTCT**CC**	64
Cr-tiar_gRNA1Fw	**AGATGG**AACTGGGCTGCAAGGAAGGG	64

Cr-tiar_gRNA1Rv	**AAAC**CCCTTCCTTGCAGCCCAGTT**CC**	64
Cr-tiar_gRNA2Fw	**AGATGG**CAAGGTGATAGTCAACCCAA	60
Cr-tiar_gRNA2Rv	**AAAC**TTGGGTTGACTATCACCTTG**CC**	60
Cr-tiar_gRNA3Fw	**AGATGG**CAGTATGGATCACCACCAGC	63
Cr-tiar_gRNA3Rv	**AAAC**GCTGGTGGTGATCCATACTG**CC**	63
Cr-ttp_gRNA1Fw	**AGATGG**TGTCGTACGTATCACACCAG	61
Cr-ttp_gRNA1Rv	**AAAC**CTGGTGTGATACGTACGACA**CC**	61
Cr-ttp_gRNA2Fw	**AGATGG**CTGTTGGATGGCGAGTAAGG	63
Cr-ttp_gRNA2Rv	**AAAC**CCTTACTCGCCATCCAACAG**CC**	63
Cr-ttp_gRNA3Fw	**AGATGG**GATTCTCGGTCGGAATCCGG	64
Cr-ttp_gRNA3Rv	**AAAC**CCGGATTCCGACCGAGAATC**CC**	64
Cr-ttp_gRNA4Fw	**AGATGG**CTTCGCTAAAAGCTCTGCTA	60
Cr-ttp_gRNA4Rv	**AAAC**TAGCAGAGCTTTTAGCGAAG**CC**	60
Cr-g3bp_cleavage1Fw	GTAGCTATGGGGGTTGAGACT	58
Cr-g3bp_cleavage1Rv	CCATCTGAGGAGAGCTGTTATG	58
Cr-g3bp_cleavage2Fw	GCATAACAGCTCTCCTCAGATG	58
Cr-g3bp_cleavage2Rv	CGCCGATGCAGTTATCCA	55
Cr-g3bp_cleavage3Fw	GAGTGCTTCATCCTGATTGTTG	56
Cr-g3bp_cleavage3Rv	CGACTGCAAGTTGCAATGC	55
Cr-tiar_cleavage1Fw	CCATGGCAGCATTATCATGC	55
Cr-tiar_cleavage1Rv	GTATTACCACTACCAGCCAGA	56

Cr-tiar_cleavage2Fw	GAGCAAGCAAATAAGGGGAAG	56
Cr-tiar_cleavage2Rv	TAGGGACCACATATGCCAT	53
Cr-tiar_cleavage3Fw	CCAGCTTAACAGGGCTACCCT	60
Cr-tiar_cleavage3Rv	ACGCGCCAAGCAAATGCA	55
Cr-ttp_cleavage1,2,4Fw	CGCCGCCACTCTTCATCTATG	60
Cr-ttp_cleavage1,2,4Rv	TACCGGGACTGACGTTTGC	57
Cr-ttp_cleavage3Fw	TACCGGGACTGACGTTTGC	57
Cr-ttp_cleavage3Rv	AGACAGGCATCGGAAGATTGG	58
FoxD-as9-in fusion-F	**TCACGAGGCCCTTACGTA**TATAGCGGTTTTGAAGTCG	75
FoxD-Cas9-in fusion-R	**TCTTTTTGGGGCTAGCCAT**CATCATCACACAACGGATT	75
Cr-g3bp_PCRFw	GGCAGATACTGTAATGGGG	56
Cr-g3bp_PCRRv	GGTTGTCAGGGTAACGAAC	55
Cr-g3bp_RTFw	TCTACCATCGGAAGCGGGA	63
Cr-g3bp_RTRv	CAGGACCAGGAACTGTTTCGTC	62
Cr-tiar_RTFw	AGGAAGGGAGGTCAAACAGG	59
Cr-tiar_RTRv	CCGGGAAGACTCGAATTTCC	58
Cr-ttp_RTFw	GCAAATACGGCGACAAATGC	59
Cr-ttp_RTRv	TAGCAATGCCAACGTCCTCT	59
Cr-βact_RTFw	GAGGTTATTCCTTCACCACCAC	62
Cr-βact_RTRv	GAGAGAACGGTGTTGGCGTA	65

Supplementary Table 2. Percentages of identity and similarity obtained by comparing Cr-G3BP2 amino acid sequence with orthologous sequences of metazoans. E-values, as well as GeneBank (*Branchiostoma floridae, Latimeria chalumnae, Patella vulgate*) and ANISEED accession numbers, are also reported.

**Species**	**Accession numbers**	**% identity**	**% similarity**	***E*-values**
Ciona savignyi	Cisavi.CG.ENS81.R27.315248-322198.14078.t	78.4	90.1	2.9e-107
Phallusia mammillata	Harore.CG.MTP2014.S200.g14562.02.t	61.0	76.7	4.9e-76
Halocynthia roretzi	Harore.CG.MTP2014.S200.g14562.02.t	49.2	66.9	2e-58
Botrylloides leachii	Boleac.CG.SB_v3.S147.g02876.01.t	48.9	69.8	1.4e-59
Branchiostoma floridae	XP_035668730.1	47.4	68.0	1.8e-40
Latimeria chalumnae	XP_006010102.1	51.6	73.1	1.3e-49
Patella vulgata	XP_050394320.1	46.4	67.5	2.3e-63
